# Perceptions of health risks of cigarette smoking: A new measure reveals widespread misunderstanding

**DOI:** 10.1371/journal.pone.0182063

**Published:** 2017-08-14

**Authors:** Jon A. Krosnick, Neil Malhotra, Cecilia Hyunjung Mo, Eduardo F. Bruera, LinChiat Chang, Josh Pasek, Randall K. Thomas

**Affiliations:** 1 Department of Communication, Stanford University, Stanford, California, United States of America; 2 Graduate School of Business, Stanford University, Stanford, California, United States of America; 3 Department of Political Science, Vanderbilt University, Nashville, Tennessee, United States of America; 4 Hoover Institution, Stanford University, Stanford, California, United States of America; 5 U.S. Department of Treasury, Washington, D.C., United States of America; 6 LinChiat Chang Consulting, LLC, San Francisco, California, United States of America; 7 Department of Communication Studies, University of Michigan, Ann Arbor, Michigan, United States of America; 8 GfK Custom Research North America, New York City, New York, United States of America; Legacy, Schroeder Institute for Tobacco Research and Policy Studies, UNITED STATES

## Abstract

Most Americans recognize that smoking causes serious diseases, yet many Americans continue to smoke. One possible explanation for this paradox is that perhaps Americans do not accurately perceive the extent to which smoking increases the probability of adverse health outcomes. This paper examines the accuracy of Americans’ perceptions of the absolute risk, attributable risk, and relative risk of lung cancer, and assesses which of these beliefs drive Americans’ smoking behavior. Using data from three national surveys, statistical analyses were performed by comparing means, medians, and distributions, and by employing Generalized Additive Models. Perceptions of relative risk were associated as expected with smoking onset and smoking cessation, whereas perceptions of absolute risk and attributable risk were not. Additionally, the relation of relative risk with smoking status was stronger among people who held their risk perceptions with more certainty. Most current smokers, former smokers, and never-smokers considerably underestimated the relative risk of smoking. If, as this paper suggests, people naturally think about the health consequences of smoking in terms of relative risk, smoking rates might be reduced if public understanding of the relative risks of smoking were more accurate and people held those beliefs with more confidence.

## Introduction

Despite a constant flow of messages reminding Americans of the health risks of cigarette smoking, and despite a steady decline in the proportion of Americans who smoke during the last 50 years, more than 20% of Americans continue to smoke regularly today [1]. This paper explores whether the continued prevalence of smoking may, in part, stem from a failure to acknowledge these risks. At first blush, this assertion may seem patently implausible; much research indicates that increasingly large proportions of Americans recognize the various dangers of smoking, and some studies even suggest that most Americans overestimate the proportion of smokers who suffer from certain smoking-related ailments [2]. Nonetheless, it is possible that people underestimate the magnitude of some of the health risks caused by smoking. Because individuals seem to base their decisions about whether to smoke on how they believe the act of smoking changes the risk of contracting specific diseases, correcting any underestimation of risk may yield future reductions in smoking onset and increases in cessation [3]. To explore these possibilities, we conducted three studies of national samples of American adults documenting risk perceptions and their relations to smoking behavior.

## Challenges in the study of risk perception

One way to gauge the accuracy of people’s perceptions of the health dangers of smoking is to focus simply on the list of maladies that become more likely as a result of smoking. This list includes various cancers, heart diseases, respiratory diseases, premature death, and more [4,5]. By asking representative national samples of American adults to identify which diseases and medical conditions on a provided list are linked with smoking, researchers have illuminated three interesting patterns. First, since the 1950s, the proportion of Americans who failed to identify any health risks of smoking dropped consistently [6]. Second, according to Gallup [7], a sizable proportion of Americans still fails to recognize a link between smoking and some related ailments (see S1 Fig). Other contemporary surveys support these same conclusions [8–10]. The proportion of American adults who associate smoking with a particular ailment varies considerably across ailments, from a high of 81% who report a link between smoking and cancer to single-digit proportions who identify links with asthma, hypertension, bronchitis, and stroke [11]. Thus, even today, Americans apparently underestimate the breadth of the danger.

A more refined way to gauge the accuracy of perceptions is to focus on the amount of increased risk of each malady that results from smoking. According to epidemiological studies, each of these increases is a function of many attributes, including age of smoking onset, number of years of regular smoking, number of cigarettes consumed per day, and more [4,5]. Therefore, actual risks must be expressed as variables that are functions of such factors, and perceptions of these risks must be ascertained specifying such factors.

Furthermore, even holding constant age of onset, length of smoking, and dosage, a smoking-related risk can be perceived in three different ways: (1) absolute risk (i.e., “what is the chance that a person will get lung cancer if he/she smokes?”), (2) attributable risk (i.e., “how much does smoking raise the chances that a person will get lung cancer compared to not smoking?”), and (3) relative risk (i.e., “how much more likely is a person to get lung cancer if he/she smokes?”) [12,13]. Mausner and Bahn [14] provide a thorough review of how epidemiologists calculate and use each of these different measures of risk. Assessing Americans’ perceptions of all three seems most sensible in order to determine whether people tend to perceive all types of risk accurately, overestimate all types of risk, underestimate all types of risk, or overestimate some while underestimating others. Attributable fraction is another measure of risk perceptions, but we do not investigate this measure in this study [15].

One way to think about the goal of such an investigation is to identify any ways in which people underestimate risk, so that public health education campaigns can correct this misunderstanding. But it could turn out that people underestimate one particular type of risk (e.g., absolute risk) and yet do not use that particular perception of risk in their decision-making about whether to start or stop smoking. Therefore, efforts to correct the public’s misunderstanding would not translate into changes in smoking behavior. So to draw out implications of measurements of perceived risk, we need evidence indicating which perceptions may be behaviorally consequential.

The research described in this paper set out to do so by gauging perceptions of absolute risk, attributable risk, and relative risk with a focus specifically on lung cancer. And we explored which of these risk perceptions might drive smoking onset and cessation. We focus on lung cancer specifically rather than all health risks associated with smoking following Viscusi’s seminal work on smoking-related risks [2]. While the share of American adults who associate smoking with a particular health malady varies across maladies [11], an assessment of which type of risk perception—absolute risk, attributable risk, and relative risk—impacts Americans’ smoking behavior the most should not be sensitive to the health malady of interest. In other words, if perceptions of relative risk of lung cancer affects smoking behavior more than perceptions of absolute and attributable risk of lung cancer, then perceptions of relative risk of another disease should similarly be most effective at driving smoking behavior.

## Prior studies of perceptions of the magnitude of risk

A number of past studies have attempted to measure perceptions of the magnitude of the risk of smoking in representative samples of American adults, but their methodologies entailed a series of limitations, as we outline next. It is worth noting that this paper focuses on the U.S. and therefore does not discuss the many interesting studies of smoking-related risk perceptions that have been done in countries other than the U.S [16–18].

We also do not discuss studies that examined people’s perceptions of their own personal smoking-related risks (e.g., Boney-McCoy et al. [19]; Strecher et al. [20]) because our focus is on Americans’ perceptions of the risk of smoking to people in general. Many studies have produced interesting results involving people’s perceptions of their own personal risks of smoking-related health problems (e.g., [19,21–27]). However, according to Gigerenzer [28], people naturally think about the population rather than personal chance, and perceptions of personal risk likely mediate the relationship between general risk and behavior.

Because this paper is focused on the beliefs of adults, we also do not discuss the findings of many interesting studies of youth. For example, Romer and Jamieson [29] asked questions similar to Viscusi’s [2] of a national sample of 14- and 15-year-olds: “Out of every 100 cigarette smokers, how many do you think will: (a) get lung cancer because they smoke? (b) have heart problems, like a heart attack, because they smoke? (c) die from a smoking-related illness?” Their results mirror Viscusi’s [2]: on average; respondents said 61.4% of smokers would develop lung cancer, much higher than the true rate. Likewise, a representative sample of 20–22 year olds said 52.6% on average. Many other studies have explored the beliefs of children and adolescents as well [21,30–37].

Some past studies have asked people to describe their perceptions of the magnitude of a smoking-related risk of some malady by asking people to select a point on a rating scale with a small number of verbally labeled response options. For example, Weinstein et al. [27] asked “How likely do you think it is that (the average male cigarette smoker/the average female cigarette smoker/you) will develop lung cancer in the future?” and offered a 5-point scale ranging from “very low” to “very high.” Similarly, Romer and Jamieson [29] asked respondents “In your opinion, is smoking very risky for a person’s health, somewhat risky, only a little risky, or not risky at all?” It is not clear whether “somewhat risky” or “very risky” is an overestimate or underestimate of risk. In other words, measures that assess perceptions of smoking’s dangers on these non-numeric subjective probability scales do not permit assessing the degree to which magnitudes of perceived risk reflect true numeric risk levels.

Other studies have measured perceptions of risks quantitatively but did not specify the population of people being described or the dosage of smoking being addressed. For example, in a survey conducted by Audits & Surveys Worldwide, respondents were asked, “Among 100 cigarette smokers, how many of them do you think will get lung cancer because they smoke?” [2]. The characteristics of a smoker are important contextual considerations with regards to actual health risks a given smoker faces. The probabilities of various smoking-related ailments differ for occasional and daily smokers and depend on the age of a smoker as well as the duration of smoking. Because this type of question does not specify what population is to be described or how much smoking was done for how long, it is impossible to gauge the accuracy of responses by comparing them with the results of epidemiological studies, which show risk to vary across populations and age, smoking duration, and dosage. Some scholarly work has begun to remedy this issue, specifying the exact quantity of cigarettes smoked per day [38].

Another potential limitation of the Audits & Surveys question is the phrase “because they smoke.” This phrase was presumably meant to lead respondents to estimate the number of lung cancer cases completely attributable to smoking. As Slovic [36] observed, this phrase can be interpreted in various different ways. Specifically, people may believe that smoking, along with other factors, enhances the chances of contracting lung cancer, leading them to respond that smoking is partially responsible for some lung cancer cases. This, too, makes it difficult to identify the appropriate true rate of smoking-induced lung cancer cases to which to compare risk perceptions.

Finally, the notions of “subadditivity” and “the focus of judgment effect” point to another potential problem with the Audits & Surveys question [39–41]. The question, “Among 100 cigarette smokers, how many of them do you think will get lung cancer because they smoke?” focuses respondents’ attention on just one possible outcome of smoking: *getting* lung cancer. This approach typically leads to overestimation of the probability of the event in question. Asking respondents instead to report the number of smokers who will *not* get lung cancer would focus attention on that outcome instead, probably leading to overstatement of that probability. So the sum of the average answers to these two forms of the question would most likely total more than 100. A more desirable measurement approach would overcome the bias induced by arbitrarily asking about only one outcome (e.g., either getting lung cancer or not getting lung cancer).

## The present research

To overcome the limitations of past studies, we conducted three surveys measuring Americans’ beliefs about smoking-related health risks in different ways. To gauge perceived risk, we asked two questions: one about the risk to nonsmokers, and the other about the risk to smokers. This approach is advantageous if a researcher wants to measure perceptions of attributable risk or relative risk, because (1) subadditivity is likely to bias both reports upward, so subtracting or dividing one judgment from or by the other will minimize the impact of overestimation, (2) answers to these questions can be used to generate assessments of perceived absolute risk, attributable risk, and relative risk, and (3) this approach employs the principle of decomposition, which enhances the accuracy of measures of people’s beliefs [15]. It is worth noting one limitation of our research is the fact that we only ask about lung cancer, and do not consider other health risks linked with smoking. However, most likely people’s perceptions of risk across multiple disease categories would be positively correlated. Consequently, our general conclusions about lung cancer would likely be similar if respondents were forced to consider multiple disease categories.

In decomposition, a single, global judgment is broken down into a series of sub-judgments, each of which a respondent must make in the process of generating the global judgment. Here, in order to gauge people’s perceptions of relative risk, we could ask, “how many more times likely is a smoker to get lung cancer than a nonsmoker?” To answer the global question, a respondent must estimate both the likelihood a nonsmoker will get lung cancer and estimate the likelihood that a smoker will get lung cancer, and then mentally compute the ratio of the probabilities. Because respondents can accidentally make a computational error when executing that last step, surveyors can more accurately measure people’s beliefs by asking directly about the sub-judgments, leaving the researcher to compute the ratio. The same logic applies to the measurement of perceived attributable risk (see S1 Appendix for a discussion of measuring probabilities and numeracy).

When measuring perceptions of the lung cancer risks of nonsmokers and smokers, we expressed specifically a volume of smoking and at what age it began, so we could more accurately gauge the extent to which people overestimated or under-estimated the health risks of smoking. And rather than asking survey respondents to report probabilities, we asked them to report frequencies, since a variety of studies suggest that people think more naturally in terms of frequencies [42,43].

We compared the three risk perception measures (absolute, attributable, and relative risk) in terms of their associations with cessation among a sample of current and former smokers. We also compared the risk perception measures in terms of their associations with the desire to quit among current smokers. Although previous studies have found positive and significant correlations between risk perceptions and the desire to quit, none of these studies compared different risk perception measures to one another or analyzed numerical risk estimates [27,44,45].

Such associations can occur for at least two reasons. First, beliefs about the health risks of smoking may be instigators of smoking cessation (for a review of this literature, see S2 Appendix). Second, people may adjust their beliefs about smoking’s health risks in order to rationalize their status as a smoker or a non-smoker [46–48]. If perceptions of health risks are motivators of smoking cessation and/or if quitting smoking induces people to inflate their risk perceptions, then perceived risk should be lower among people who currently smoke than among people who have quit. That is, the higher a person’s perceived risk, the more likely he or she is to have quit. Likewise, the higher a current smoker’s perception of risk, the more motivated he or she should be to quit smoking. Therefore, the more strongly a risk perception measure is associated with whether a person has quit smoking and a smoker’s desire to quit, the more likely that risk perception is to capture the way people naturally think about risk in this arena.

Many possible patterns of risk perception types could be found in a population. The most heterogeneous pattern would be one in which one-third of people think in terms of absolute risk, while another one-third of people think in terms of attributable risk, and the remaining people think in terms of relative risk. The most homogeneous case would be one in which everyone thinks in terms of just one of these risk perceptions to make behavioral choices regarding smoking. Our analyses explored the extent of use of each of the three risk perception measures.

We also explored whether people who felt more certain about risk perceptions manifested stronger relations of those perceptions with cessation and desire to quit. Psychological research on attitude strength suggests that people hold beliefs and attitudes with varying degrees of certainty, and beliefs held with more certainty are more likely to shape thinking and action [49]. Therefore, we explored whether any of the risk perceptions were more strongly related to cessation among people who held their risk perceptions with more certainty.

## Three studies

Our three studies explored five main questions: (1) How many people overestimate and underestimate absolute risk, attributable risk, and relative risk of lung cancer due to smoking? (2) How strongly are perceived absolute risk, attributable risk, and relative risk related to quitting? (3) How strongly are perceived absolute risk, attributable risk, and relative risk related to desire to quit among current smokers? (4) Are the relations between risk perceptions and quitting strongest among respondents who are most certain about their risk perceptions? (5) How strongly are perceived absolute risk, attributable risk, and relative risk related to having initiated smoking?

Study 1 was a random digit dial telephone survey of a nationally representative sample of American adults who were current or former smokers, conducted in 2000 by Schulman, Ronca, and Bucuvalas, Inc. (hereafter SRBI). Study 2 was a 2006 survey of a national non-representative sample of current and former smokers who volunteered to complete Internet surveys for Harris Interactive in exchange for points that could be redeemed for gifts. Study 3 was a 2009 survey of a nationally representative sample of all Americans, including people who had never smoked, via the Face-to-Face Recruited Internet Survey Platform (the FFRISP; see S3 Appendix for descriptions of the methodologies of the three studies, and see S4 Appendix for the demographic characteristics of the three samples).

The telephone survey respondents who were current or former smokers were asked:

(1) “Next, I'd like to turn to a different topic: what you personally think about the effect of cigarette smoking on people's health. I'm going to read these next two questions very slowly to let you think about each part of them, and I can repeat each question as many times as you like before you answer, so you can be sure they are clear to you. First, if we were to randomly choose one thousand American adults who never smoked cigarettes at all during their lives, how many of those one thousand people do you think would get lung cancer sometime during their lives?”

(2) “And if we were to randomly choose one thousand American adults who each smoked one pack of cigarettes a day every day for 20 years starting when they were 20 years old, how many of those one thousand people do you think would get lung cancer sometime during their lives?”

(3) “You said that smokers are [more likely/as likely/less likely] to get lung cancer than nonsmokers. How certain are you about this? Extremely certain, very certain, moderately certain, slightly certain, or not certain at all?”

We ask respondents to assess the prospect of lung cancer incidence generally like Viscusi [2]. We emphasized “personally” so that people would feel comfortable providing their own best guess of a fact, specifically general population risk of contracting lung cancer. This wording is designed to avoid the question seeming like a “quiz” (or their guess of what a public health authority might say), but rather their personal assessment of risk. For the two Internet surveys, the wording was adapted for self-administration. In all three studies, the response choices for the last question were presented in descending order for a randomly chosen half of the respondents and in ascending order for the other half. By implementing the same internally valid research design three separate times, it is possible to assess whether our findings are replicable.

Each of the three studies discussed above were deemed as suitable for exempt IRB review status by Stanford University’s review board, as no identifying information on the respondents was retained, and disclosure of answers to the survey questions would not place the respondents at risk. Informed consent for Study 1 was provided verbally given that Study 1 was a telephone survey. Written informed consent was provided for both Study 2 and Study 3, and Stanford’s IRB approved use of oral consent in Study 1 and written consent in Study 2 and 3.

### Actual risk

We used data reported by Peto et al. [50] to compute the actual absolute risk, attributable risk, and relative risk of contracting lung cancer for one-pack-a-day smokers who started smoking at age 20 and smoked for 20 years. To do so, we divided the absolute risk of mortality due to lung cancer among these smokers (about 3%) by the absolute risk of mortality due to lung cancer among non-smokers (about 0.4%, yielding a relative risk of about 7). Although Peto et al. [50] examined mortality instead of incidence, the probability of dying from lung cancer conditional on developing lung cancer is 74.4% within a thirteen-year period according to Marcus et al. [51], and even higher among smokers [52]. If relative risk is higher, then our results understate the proportion of Americans who underestimate this relative risk. According to these figures, the attributable risk of lung cancer due to smoking is then about 3% (3% minus 0.4%, rounds to 3%). It is worth noting that although one might imagine that it is difficult to estimate risk rates because of complex functional forms, interactions of smoking with other risk factors, cohort effects, and other complications, research suggests that in fact, risk rates are largely robust to some potential complexities [53–55].

### Perceived risk

In Study 1, the mean of current and former smokers’ perceptions of absolute risk of lung cancer among smokers was 48% (i.e., 480.1 smokers out of 1,000 smokers would get lung cancer); the median was 50% (see columns 1 and 2 of Table 1). 10.3% of respondents perceived absolute risks between 0% and 5.0%, and the remaining respondents gave answers above 5.0%. 99.5% of respondents overestimated absolute risk, only about 0.3% estimated it correctly (by giving an answer of 30), and 0.2% underestimated it (by giving an answer less than 30).

**Table 1:** Perceived numbers of nonsmokers and smokers who will get lung cancer: SRBI, Harris Interactive, and FFRISP Surveys.

	Distribution of Responses (SRBIS Survey)–Current and Former Smokers		Distribution of Responses After Removing Respondents Who Responded 500 on Either Risk Perception Question (SRBI Survey)–Current and Former Smokers		Distribution of Responses After Imputing Risk Perceptions for Respondents Who Responded 500 on Either Question (SRBI Survey)–Current and Former Smokers		Distribution of Responses (Harris Interactive Survey)–Current and Former Smokers		Distribution of Responses (FFRISP Survey)–Current and Former Smokers		Distribution of Responses (FFRISP Survey)–Never Smokers	
Number of People Who Would get Lung Cancer	Out of 1,000 nonsmokers	Out of 1,000 smokers	Out of 1,000 nonsmokers	Out of 1,000 smokers	Out of 1,000 nonsmokers	Out of 1,000 smokers	Out of 1,000 nonsmokers	Out of 1,000 smokers	Out of 1,000 nonsmokers	Out of 1,000 smokers	Out of 1,000 nonsmokers	Out of 1,000 smokers
0–50	36.0%	10.3%	44.0%	14.8%	36.9%	11.7%	49.5%	25.7%	53.2%	25.4%	55.5%	17.9%
51–100	17.6%	8.0%	20.5%	11.5%	18.3%	8.6%	16.4%	13.5%	17.0%	13.5%	21.1%	12.7%
101–150	2.4%	1.5%	3.1%	2.2%	6.3%	3.5%	2.4%	2.2%	2.0%	1.6%	3.3%	0.6%
151–200	9.3%	5.1%	10.0%	6.5%	12.6%	6.2%	5.7%	7.5%	9.9%	4.6%	7.5%	4.0%
201–250	4.3%	2.1%	3.9%	3.1%	6.1%	4.1%	4.7%	3.5%	3.0%	4.1%	1.8%	1.9%
251–300	2.5%	4.8%	3.2%	6.9%	3.8%	5.7%	2.9%	4.4%	5.6%	8.0%	2.3%	6.0%
301–350	2.2%	0.8%	2.7%	1.1%	4.2%	3.5%	0.7%	1.6%	0.7%	1.1%	0.6%	2.3%
351–400	2.4%	8.3%	3.1%	10.1%	3.1%	9.5%	2.2%	3.0%	2.5%	4.8%	1.1%	5.7%
401–450	0.2%	2.4%	0.3%	3.4%	1.6%	4.1%	0.3%	0.4%	0.0%	0.4%	0.0%	0.9%
451–499	0.0%	0.0%	0.0%	0.0%	0.0%	1.0%	0.0%	0.1%	0.1%	0.0%	0.1%	0.3%
500	16.4%	19.1%	0.0%	0.0%	0.0%	0.0%	10.1%	15.5%	4.3%	15.5%	3.3%	9.5%
501–550	0.0%	0.9%	0.0%	1.3%	0.0%	1.9%	0.0%	0.8%	0.1%	0.5%	0.1%	0.2%
551–600	1.0%	4.9%	0.9%	6.2%	1.3%	6.4%	0.8%	2.9%	1.4%	3.4%	0.9%	6.8%
601–650	0.5%	0.6%	0.7%	0.8%	0.5%	1.7%	0.4%	0.7%	0.0%	0.9%	0.2%	1.0%
651–700	1.0%	6.9%	1.5%	6.1%	1.0%	7.6%	0.9%	4.6%	0.0%	3.2%	0.3%	5.8%
701–750	1.1%	5.2%	1.4%	5.0%	1.1%	5.6%	0.4%	2.3%	0.0%	2.7%	0.3%	4.1%
751–800	2.1%	6.2%	3.1%	6.9%	2.1%	6.6%	1.0%	2.9%	0.0%	2.6%	0.5%	5.3%
801–850	0.0%	1.2%	0.0%	1.7%	0.0%	1.2%	0.2%	0.4%	0.1%	1.7%	0.0%	1.5%
851–900	0.4%	5.5%	0.6%	5.0%	0.4%	5.7%	0.5%	4.6%	0.0%	3.1%	0.5%	5.9%
901–950	0.0%	0.8%	0.0%	1.2%	0.0%	0.8%	0.0%	0.5%	0.0%	0.2%	0.0%	2.0%
951–1000	0.7%	5.5%	0.9%	6.2%	0.7%	4.9%	1.0%	2.8%	0.1%	2.8%	0.5%	5.6%
Total	100.0%	100.0%	100.0%	100.0%	100.0%	100.0%	100.0%	100.0%	100.0%	100.0%	100.0%	100.0%
Mean	215.5	480.1	165.5	435.2	170.1	448.5	164.6	332.6	119.2	330.7	110.4	433.2
Median	100	500	100	400	100	400	60	250	50	300	50	400
N	458	466	329	336	458	466	801	801	452	451	512	512

As expected, the mean and median perceived absolute risk of nonsmokers getting lung cancer were less: 22% and 10%, respectively. Thirty-six percent of respondents gave answers between 0% and 5.0%. Thus, most people vastly overestimated this absolute risk.

Only 5.2% of respondents thought smokers were less likely to get lung cancer than nonsmokers (a belief revealed by attributable risks less than 0; see columns 1 and 2 of Table 2). Attributable risk was calculated by subtracting each respondent’s answer to the question about nonsmokers from his or her answer to the question about smokers. 9.6% of respondents thought smokers and nonsmokers were equally likely to contract lung cancer, reporting an attributable risk of 0. A large majority, 85.2% of respondents, reported that smokers were more likely than nonsmokers to contract lung cancer. 76.1% overestimated attributable risk by reporting figures greater than 4%. The mean perceived attributable risk was about 27%, and the median was 20%.

**Table 2:** Perceived attributable risk of getting lung cancer from cigarette smoking: SRBI, Harris Interactive, and FFRISP Surveys.

	Distribution of Responses (SRBIS Survey)–Current and Former Smokers		Distribution of Responses After Removing Respondents Who Responded 500 on Either Risk Perception Question (SRBI Survey)–Current and Former Smokers		Distribution of Responses After Imputing Risk Perceptions for Respondents Who Responded 500 on Either Question (SRBI Survey)–Current and Former Smokers		Distribution of Responses (Harris Interactive Survey)–Current and Former Smokers		Distribution of Responses (FFRISP Survey)–Current and Former Smokers		Distribution of Responses (FFRISP Survey)–Never Smokers	
Perceived Attributable Risk	Valid percent	Cumulative Percent	Valid percent	Cumulative Percent	Valid percent	Cumulative Percent	Valid percent	Cumulative Percent	Valid percent	Cumulative Percent	Valid percent	Cumulative Percent
-1,000 to -1	5.2%	5.2	4.0%	4.0	4.2%	4.2	5.3%	5.3	6.4%	6.4	3.9%	3.9
0	9.6%	14.8	7.2%	11.2	5.0%	9.2	15.0%	20.4	12.1%	18.5	6.3%	10.3
1–49	9.1%	23.9	13.2%	24.4	10.3%	19.5	20.1%	40.5	20.4%	38.8	19.1%	29.4
50–99	6.8%	30.7	9.8%	34.2	8.0%	27.6	10.4%	50.9	10.8%	49.6	8.7%	38.0
100–149	5.3%	36.0	6.5%	40.7	7.7%	35.3	5.8%	56.7	5.1%	54.7	3.3%	41.3
150–199	5.5%	41.5	7.5%	48.2	7.7%	43.0	4.2%	60.9	6.5%	61.2	4.4%	45.7
200–249	9.8%	51.2	9.8%	58.1	10.2%	53.2	6.9%	67.7	3.4%	64.6	2.5%	48.3
250–299	5.9%	57.2	3.7%	61.8	5.2%	58.4	3.9%	71.6	7.9%	72.5	7.6%	55.8
300–349	6.9%	64.0	4.3%	66.1	4.3%	62.6	6.9%	78.5	2.2%	74.7	3.1%	58.9
350–399	3.9%	68.0	5.4%	71.4	6.7%	69.3	1.8%	80.2	4.4%	79.1	5.8%	64.7
400–449	7.6%	75.5	3.0%	74.4	3.6%	72.9	4.8%	85.0	3.4%	82.5	2.5%	67.2
450–499	4.3%	79.8	1.1%	75.5	3.7%	76.5	4.1%	89.2	4.1%	86.6	2.5%	69.7
500	4.6%	84.4	1.9%	77.4	1.3%	77.8	2.7%	91.9	1.7%	88.3	3.6%	73.3
501–549	0.3%	84.7	0.4%	77.8	1.1%	78.9	0.6%	92.4	0.7%	89.1	2.3%	75.7
550–599	2.2%	86.9	3.2%	81.0	4.6%	83.5	1.0%	93.5	2.6%	91.7	5.2%	80.9
600–649	2.2%	89.2	3.3%	84.3	3.2%	86.6	0.2%	93.7	1.7%	93.3	2.0%	82.9
650–699	1.4%	90.5	2.0%	86.3	2.4%	89.1	1.8%	95.5	1.9%	95.2	3.5%	86.4
700–749	3.0%	93.5	4.3%	90.6	4.0%	93.0	1.2%	96.7	0.9%	96.1	1.7%	88.0
750–799	1.9%	95.4	2.8%	93.3	2.1%	95.1	1.0%	97.6	1.3%	97.4	3.2%	91.2
800–849	2.0%	97.4	2.9%	96.2	2.3%	97.4	1.2%	98.8	0.0%	97.4	1.4%	92.6
850–899	1.3%	98.7	1.9%	98.1	1.3%	97.7	0.7%	99.6	1.6%	98.9	3.3%	95.9
900–949	0.0%	98.7	0.0%	98.1	0.0%	98.7	0.2%	99.7	0.2%	99.2	1.8%	97.7
950–1,000	1.3%	100.0	1.9%	100.0	1.3%	100.0	0.3%	100.0	0.8%	100.0	2.3%	100.0
Total	100.0%	100.0	100.0%	100.0	100.0%	100.0	100.0%	100.0	100.0%	100.0	100.0%	100.0
Mean	267.4	-	272.4	-	289	-	168	-	211.3	-	320.1	-
Median	200	-	200	-	225.4	-	95	-	115	-	280	-
N	456	-	328	-	456	-	801	-	451	-	511	-

In contrast, a large majority of respondents (74.6%) underestimated relative risk, because they reported perceptions that implied a relative risk less than 7 (see columns 1 and 2 of Table 3). Relative risk was computed by dividing each respondent's answer to the question about 1,000 smokers by his or her answer to the question about 1,000 nonsmokers. Because this quantity is undefined for respondents who said none of the 1,000 nonsmokers would get lung cancer (because the denominator would be zero), 1 was added to these respondents’ answers to the questions about smokers and nonsmokers to allow the relative risk quantity to be defined for all respondents. Note that re-computing all analyses reported below treating these people as having missing data on the relative risk measure had negligible impact on the reported results. 54.6% of the respondents could be said to have vastly underestimated relative risk, because their reports implied a value less than 3. Only about 1.5% of respondents perceived relative risk approximately correctly (e.g., 7), and only 23.9% of respondents overestimated relative risk. 5.2% of respondents perceived a relative risk of less than 1, meaning they thought smokers developed lung cancer less often than nonsmokers, and 9.6% of the sample perceived a relative risk of 1.0, meaning they thought smokers and nonsmokers were equally likely to develop lung cancer. Mean perceived relative risk was 26.7, much higher than the true value, and the median was 2.5, lower than the true value. Thus, relative risk tells a very different story about the prevalent errors in risk perceptions than does attributable risk: most people overestimated the latter, whereas most people underestimated the former.

**Table 3:** Perceived relative risk of getting lung cancer from cigarette smoking: SRBI, Harris Interactive, and FFRISP Surveys.

	Distribution of Responses (SRBIS Survey)–Current and Former Smokers		Distribution of Responses After Removing Respondents Who Responded 500 on Either Risk Perception Question (SRBI Survey)–Current and Former Smokers		Distribution of Responses After Imputing Risk Perceptions for Respondents Who Responded 500 on Either Question (SRBI Survey)–Current and Former Smokers		Distribution of Responses (Harris Interactive Survey)–Current and Former Smokers		Distribution of Responses (FFRISP Survey)–Current and Former Smokers		Distribution of Responses (FFRISP Survey)–Never Smokers	
Perceived Relative Risk	Valid percent	Cumulative Percent	Valid percent	Cumulative Percent	Valid percent	Cumulative Percent	Valid percent	Cumulative Percent	Valid percent	Cumulative Percent	Valid percent	Cumulative Percent
.001-.99	5.2%	5.2	4.0%	4.0	4.2%	4.2	5.3%	5.3	6.4%	6.4	3.9%	3.9
1	9.6%	14.8	7.2%	11.2	5.0%	9.2	15.0%	20.4	12.1%	18.5	6.3%	10.3
1.01–1.99	28.4%	43.3	23.4%	34.7	21.7%	30.9	28.4%	48.8	20.1%	38.6	14.2%	24.5
2.00–2.99	11.4%	54.6	13.0%	47.7	17.2%	48.1	10.8%	59.5	18.3%	56.9	10.7%	35.2
3.00–3.99	8.1%	62.7	11.8%	59.5	12.2%	60.3	8.4%	67.9	9.1%	66.0	10.2%	45.4
4.00–4.99	6.9%	69.6	4.7%	64.2	6.4%	66.7	6.9%	74.8	5.3%	71.3	7.9%	53.3
5.00–5.99	2.5%	72.1	3.6%	67.8	4.4%	71.1	3.4%	78.3	3.5%	74.8	3.7%	57.0
6.00–6.99	2.5%	74.6	3.6%	71.3	3.1%	74.2	1.2%	79.5	2.4%	77.2	2.7%	59.7
7.00–7.99	2.9%	77.5	4.2%	75.5	3.4%	77.7	1.5%	80.9	3.0%	80.3	3.6%	63.4
8.00–8.99	2.1%	79.6	3.1%	78.6	2.3%	80.0	2.0%	83.0	1.0%	81.2	2.8%	66.1
9.00–9.99	1.8%	81.4	0.9%	79.5	1.4%	81.4	2.4%	85.4	2.7%	83.9	6.2%	72.3
10.00–10.99	0.2%	81.6	0.2%	79.7	0.3%	81.7	0.9%	86.3	0.2%	84.1	1.3%	73.6
11.00–11.99	0.7%	82.2	1.0%	80.7	0.7%	82.3	0.4%	86.7	1.0%	85.1	1.5%	75.1
12.00–12.99	0.7%	82.9	1.0%	81.7	1.2%	83.6	0.9%	87.6	0.6%	85.7	0.7%	75.9
13.00–13.99	0.0%	82.9	0.1%	81.7	0.0%	83.6	1.1%	88.7	0.4%	86.1	0.8%	76.7
14.00–14.99	1.2%	84.2	1.6%	83.3	1.1%	94.7	0.6%	89.3	0.8%	86.9	2.1%	78.8
15.00–15.99	0.2%	84.4	0.3%	83.6	0.9%	85.6	0.1%	89.4	0.2%	87.1	0.4%	79.3
16.00–16.99	1.6%	85.9	1.9%	85.5	1.3%	86.7	0.5%	90.0	0.5%	87.6	0.6%	79.8
17.00–17.99	0.8%	86.7	1.2%	86.7	1.1%	87.9	0.3%	90.3	0.4%	88.0	0.9%	80.7
18.00–18.99	1.1%	87.8	1.5%	88.2	1.1%	89.0	1.1%	91.4	0.5%	88.5	1.0%	81.7
19.00–19.99	1.1%	88.8	0.6%	88.7	0.5%	89.4	0.4%	91.8	0.7%	89.1	0.2%	81.9
20.00–29.99	1.5%	90.3	2.1%	90.8	1.7%	91.1	2.6%	94.4	3.6%	92.7	4.2%	86.1
30.00–49.99	3.0%	93.2	2.7%	93.5	2.1%	93.3	2.3%	96.7	2.8%	95.5	2.5%	88.6
50.00–79.99	1.4%	94.6	2.0%	95.5	2.0%	95.3	1.2%	97.9	1.6%	97.1	4.3%	92.9
80.00–1001.00	5.4%	100.0	4.5%	100.0	4.7%	100.0	2.1%	100.0	2.9%	100.0	7.1%	100.0
Total	100.0%	100.0	100.0%	100.0	100.0%	100.0	100.0%	100.0	100.0%	100.0	100.0%	100.0
Mean	26.7	-	21.2	-	18.4	-	9.5	-	12.9	-	26.6	-
Median	2.5	-	3.5	-	3.3	-	2.3	-	2.5	-	4.9	-
N	456	-	328	-	456	-	801	-	451	-	511	-

Compared to the representative sample of current and formers smokers interviewed in Study 1, Study 2’s non-probability sample of current and former smokers reported: (1) lower perceived absolute risk of lung cancer among nonsmokers and smokers (e.g., 49.5% and 25.7%, respectively, gave answers between 0 and 50 out of 1,000 who would get lung cancer, compared to 36.0% and 10.3% in Study 1; see seventh and eighth columns in Table 1); (2) lower perceived attributable risk (e.g., 50.9% had a value of 99 or less, compared to 30.7% of the Study 1 respondents; see the eighth column of Table 2); and (3) lower perceived relative risk (e.g., 59.5% had values of 2.99 or less, as compared with 54.6% of the Study 1 respondents; see the eighth column of Table 3).

Using all three risk measures, Study 3’s representative sample of current and former smokers perceived less risk than the Study 1’s respondents did 9 years earlier. Study 3’s current and former smokers reported lower absolute risk among nonsmokers (mean = 11.9%, median = 5%) than did the Study 1 respondents (mean = 21.5%, median = 10%; see columns nine and one, respectively, of Table 1). Study 3’s current and former smokers perceived lower absolute risk for smokers than did the Study 1 respondents (means = 33.1% vs. 48.0%; medians = 30.0% vs. 50.0%; see columns ten and two, respectively, of Table 1). And Study 3’s current and former smokers perceived lower attributable risk of smoking than did the Study 1 respondents (means = 21.1% vs. 26.7%; medians = 11.5% vs. 20.0%; see columns nine and one, respectively, of Table 2) and lower relative risk than did the Study 1 respondents (means = 12.9 vs. 26.7; medians = 2.5 vs. 2.5; see columns 9 and 1, respectively, of Table 3).

Study 3 suggests that the perceived risk of lung cancer may have declined among current and former smokers between 2000 and 2009. That is, the two representative sample surveys indicated that respondents’ assessments of the absolute risk of lung cancer for both smokers and non-smokers became notably more accurate during this period.

### Comparing risk measures

Which of these measures is an appropriate focus for claims about public risk perceptions and their accuracy? One way to answer this question is to determine which of these risk perceptions drives people’s decisions about whether or not to smoke. Many possible patterns of risk perception use are possible in any population. The most heterogeneous pattern would be one in which some people decide whether to smoke or quit based upon their perceptions of the attributable risk, while others make this decision with reference to perceptions of relative risk, and still others make their decisions based on perceptions of absolute risk, with the three groups being of roughly equal size. The most homogeneous case is that in which everyone uses just one of these risk perceptions to make their behavioral choices regarding smoking. By gauging which risk perceptions have how much impact for how many people, we can begin to understand whether smoking behavior overall in a population is driven mostly by perceptions that overestimate risk, mostly by perceptions that underestimate risk, or by a mixture of perceptions that sometimes overestimate and other times underestimate.

The data of all three studies allowed us to explore whether perceptions of attributable risk, relative risk, and absolute risk inspire people to quit smoking by comparing current and former smokers. If perceptions of health risks are indeed a principal motivator of smoking cessation, then perceived risk should be lower among people who currently smoke than among people who used to smoke but have quit. In other words, the higher a person’s perceived risk, the more likely he or she should be to have quit smoking. Based upon this assumption, the better a risk perception measure predicts whether a person has quit smoking, the more likely that risk perception is to have driven quitting decisions.

To adjudicate whether absolute risk, attributable risk, or relative risk drove people’s decisions to quit, we estimated the parameters of generalized additive models (GAMs) comparing current smokers to former smokers by using a Gaussian link function predicting a binary variable representing whether a respondent was a current or former smoker using the various measures of perceived risk and the weights for unequal probability of selection and demographic post-stratification (see S5 Appendix for more details on GAMs). GAMs are especially useful for estimating models containing two highly correlated predictors (as we have here) because relaxing the assumption of linearity prevents model misspecification, allowing for better isolation of the unique relations of different risk perceptions with other variables.

Using this flexible approach, we first estimated a model in which relative and attributable risk predicted quitting (more precisely, having quit). It might seem appealing to estimate GAMs predicting quitting using all three measures, but non-independence among the three measures of perceived risk makes that impossible. When examining Study 1’s data, we see that perceptions of relative risk were sensibly correlated with diminished chances of remaining a smoker (see the top-left panel of S2 Fig). The dark line in the figure represents the estimated relation, and the two light lines demark the bounds of the 95% confidence interval around the estimates. The small vertical lines at the bottom of the figure (called “rugmarks”) indicate whether one or more respondents provided a data point at each point along the x-axis. Increasing perceived relative risk was associated with decreased log-odds of remaining a smoker. Movement from the 25^th^ percentile to the 75^th^ percentile (weighted) of relative risk increased the probability of quitting by 13.8 percentage points (see the first row of the first column of Table 4).

**Table 4:** Comparing risk measures: SRBI, Harris Interactive, and FFRISP Surveys.

Comparing the Effects of Relative Risk and Attributable Risk	Comparing the Effects of Relative Risk and Attributable Risk					
	Predicting Quitting (Current and Former Smokers)			Predicting Desire to Quit (Current Smokers)		
	SRBI	Harris	FFRISP	SRBI	Harris	FFRISP
Comparing the Effects of Relative Risk and Attributable Risk						
Effect of Relative Risk^a^	13.8%	18.4%	15.4%	17.0%	11.5%	20.0%
Effect of Attributable Risk^b^	.3%	1.6%	6.6%	-1.1%	2.6%	-7.3%
LR Test from Adding Relative Risk to Attributable Risk	.03	<.001	.006	.09	.02	<.001
LR Test from Adding Attributable Risk to Relative Risk	.64	.49	.04	.27	.08	.08
Comparing the Effects of Relative Risk and Absolute Risk						
Effect of Relative Risk^c^	15.2%	18.0%	17.4%	13.9%	11.9%	13.4%
Effect of Absolute Risk^d^	-10.5%	-1.9%	-1.1%	-15.6%	-7.2%	-0.7%
LR Test from Adding Relative Risk to Absolute Risk	.002	<.001	<.001	.05	.008	.004
LR Test from Adding Absolute Risk to Relative Risk	.15	.02	.12	.06	.02	.49

^a^Percentages indicate the increase in the predicting probability of quitting (and desire to quit) of moving from the 25th percentile of relative risk to the 75^th^ percentile of relative risk based on a GAM including both relative risk and attributable risk.

^b^Percentages indicate the increase in the predicting probability of quitting (and desire to quit) of moving from the 25th percentile of attributable risk to the 75^th^ percentile of attributable risk based on a GAM including both relative risk and attributable risk.

^c^Percentages indicate the increase in the predicting probability of quitting (and desire to quit) of moving from the 25th percentile of relative risk to the 75^th^ percentile of relative risk based on a GAM including both relative risk and absolute risk.

^d^Percentages indicate the increase in the predicting probability of quitting (and desire to quit) of moving from the 25th percentile of absolute risk to the 75^th^ percentile of absolute risk based on a GAM including both relative risk and absolute risk.

Note: In the Harris data, six outliers were removed who reported attributable risks less than or equal to -500. In the FFRISP data, five outliers were removed who reported attributable risks less than or equal to -450.

In contrast, over the range of the bulk of the data (where the majority of the rugmarks on the x-axis are located), the relation between attributable risk and quitting was fairly flat (see bottom-left panel of S2 Fig). Movement across the interquartile range of attributable risk increased the probability of quitting negligibly, by only 0.3% (see second row of the first column of Table 4).

To more formally gauge and compare these relations, we estimated a set of nested GAMs. First, we estimated a model predicting quitting using only attributable risk and then observed the improvement in goodness of fit of the model when we added relative risk as a predictor. A likelihood ratio (hereafter LR) test comparing the log likelihood of the two-variable model to the nested one-variable model indicated that the addition of the extra variable resulted in a significantly better fit (p=.03), meaning that relative risk was a reliable unique predictor of quitting (see third row of the first column of Table 4). Next, we estimated a model predicting quitting using only relative risk and then estimated the improvement in goodness of fit when attributable risk was added as a predictor. This addition did not improve the model’s fit significantly (p=.64; see fourth row of the first column of Table 4). Thus, relative risk perceptions appear to have been related to decisions to quit smoking, whereas perceptions of attributable risk were not.

To explore whether absolute risk outperforms relative risk, we estimated a GAM in which quitting was predicted by both measures. As shown in the right panels of S2 Fig, relative risk was again sensibly related to quitting (with probability of remaining a smoker declining smoothly as perceived risk increased), whereas absolute risk was not. Again, adding relative risk to a model fitted with only absolute risk improved the fit significantly (p=.002), whereas adding absolute risk to a model with relative risk did not yield a significant improvement in fit (p=.15; see rows seven and eight of the first column of Table 4). Movement across the interquartile range of absolute risk was associated with a 10.5% decrease in the chances of quitting, whereas movement across the interquartile range of relative risk was associated with a sizable and more reasonable 15.2% increase in the likelihood of quitting (see rows five and six of the first column of Table 4). As shown in columns two and three of Table 4 (as well as S3 and S4 Figs), these same results were replicated in Studies 2 and 3.

There may be an illusion hidden in these results. When people are asked to report a probability but do not know the answer, they sometimes answer “50,” meaning “fifty-fifty” or “I don’t know,” rather than meaning a 50% chance [56]. To explore the impact of this potential source of measurement error on our conclusions, we re-estimated the logistic GAM by: (1) dropping the respondents who answered “500” to the question about nonsmokers or to the question about smokers; (2) replacing the 500s with values generated by multiple imputation; and (3) replacing the 500s with answers obtained by a follow-up probe. The results supported the above conclusions even more strongly (for details of these approaches and results, see S6 Appendix).

#### Certainty

Next, we explored whether certainty moderated the associations of risk perceptions with quitting behavior. In Study 1, as expected, the correlation of relative risk with quitting was significantly stronger among high certainty respondents (people who were extremely certain, 27% of the sample) than among lower certainty respondents. Among the high certainty respondents, the probability of quitting increased over the interquartile range of relative risk by 23.7 percentage points (p=.008), a much larger increase than among the low certainty respondents, whose positive change was just 10.5 percentage points (p=.054). Accounting for certainty significantly improved the goodness of fit of the model (p=.03).

Likewise, in Study 2, the positive relation between perceived relative risk and quitting was significantly stronger among high certainty respondents than among low certainty respondents (p=.009). Among the high certainty respondents (18% of the sample), movement across the interquartile range of relative risk increased the probability of quitting by 44.1% (p<.001), whereas movement across this interquartile range in the low certainty group was associated with an increase in quitting probability of only 13.6% (p<.001). Accounting for certainty significantly improved the goodness of fit of the model (p=.009).

In Study 3, among high certainty individuals (30.5% of the sample), movement across the interquartile range of relative risk was associated with an increased probability of quitting smoking of 15.8% (p=.06), whereas movement across this interquartile range in the low certainty group was associated with an increase in quitting probability of 11.1% (p=.03). Accounting for certainty again significantly improved the goodness of fit of the model (p=.03).

#### Desire to quit

Next, we examined whether current smokers’ risk perceptions were associated with their desire to quit. While a desire to quit does not automatically translate to smoking cessation, a strong desire to quit is predictive of subsequent quitting behavior, and is a necessary condition for quitting [57]. In Study 1, adding relative risk to a GAM model predicting desire to quit among current smokers with attributable risk caused a marginally non-significant improvement in fit (p=.09; see the third row of column four in Table 4). Movement from the 25^th^ to the 75^th^ percentile of relative risk raised the probability of wanting to quit by 17.0% (see the first row of column four in Table 4). But adding attributable risk to a model predicting desire to quit with relative risk did not improve fit significantly (p=.27; see row four of column four in Table 4). Movement across the interquartile range of attributable risk slightly lowered desire to quit by 1.1% (see row two of column four in Table 4). Likewise, adding relative risk to a model including absolute risk yielded a significant improvement in fit (p=.046; see row seven of column four in Table 4). Movement across the interquartile range of relative risk increased desire to quit by 13.9% (see row five in Table 4). But adding absolute risk to a model including relative risk marginally significantly decreased desire to quit (interquartile range movement = 15.6%, p=.06; see rows six and eight of column four in Table 4). The data from Studies 2 and 3 yielded similar results (see columns five and six of Table 4). This further supports the contention that people think in terms of relative risk perceptions.

#### Smoking onset

We observed the expected results when we used the three measures in Study 3 to explore whether perceived risk was greater among people who ever smoked than among people who never smoked. Comparing the distributions in the ninth and tenth columns in Table 1 with the distributions in the last two columns of the table, we see that: (1) both groups had similar expectations for the proportion of nonsmokers who would get lung cancer (mean = 11% for people who never smoked vs. 12% for people who ever smoked), but (2) the expected proportion of smokers who would get lung cancer was higher among people who had never smoked (mean = 43.3%) than among people who ever smoked (mean = 33.1%).

Also as expected, people who never smoked perceived higher attributable risk of smoking than did people who ever smoked (see the last two columns in Table 2): (1) 3.9% thought that smokers were less likely to contract lung cancer than nonsmokers (attributable risk of less than 0); (2) 6.3% thought that smokers and nonsmokers were equally likely to get lung cancer (attributable risk of 0); and (3) 89.7% thought that smokers were more likely to contract lung cancer than nonsmokers. Respondents who never smoked thought smokers were 32 percentage points more likely than nonsmokers to get lung cancer, on average (see columns 11 and 12 of Table 2). Thus, these individuals perceived a higher attributable risk than did current and former smokers (21.1 percentage points; see column nine of Table 2). Likewise, respondents who never smoked also perceived higher relative risk than did current and former smokers (compare the last two columns of Table 3 with the ninth and tenth columns of that table).

As expected, perceptions of relative risk were strongly associated with status as a never smoker vs. a current smoker in GAMs (see the left panels of S5 Fig). Adding relative risk to a model predicting current smoking with attributable risk considerably improved fit (p<.001), whereas adding attributable risk to a model with relative risk did not significantly improve fit (p=.57). Movement across the interquartile range of relative risk yielded a 22.7 percentage point decrease in the likelihood that respondents were smokers. Movement across the interquartile range of attributable risk yielded a decrease in the probability of being a smoker of only 0.7 percentage points.

Likewise, adding relative risk to a model with only absolute risk improved fit significantly (p<.001), whereas adding absolute risk to a model including relative risk was associated with only a marginally significant improvement in fit (p=.07). Movement across the interquartile range of relative risk (when controlling for absolute risk) was associated with a 22.3 percentage point decrease in the probability of ever having smoked (see the right panels of S5 Fig). In contrast, movement across the interquartile range of absolute risk (when controlling for relative risk) produced only an 8.5 percentage point decrease in the likelihood of ever having smoked.

## Discussion

### Summary and implications

Taken together, this evidence suggests that while Americans have overestimated the absolute risk and risk difference of lung cancer associated with cigarette smoking, Americans have generally underestimated the relative risk. Furthermore, this evidence suggests that people may think more about smoking health risks in terms of relative risk than in terms of absolute risk or risk difference. The relations we saw here may result from the influence of health risk beliefs on decisions to quit smoking, decisions to start smoking, and regret about smoking, or these relations may occur because people rationalize their smoking status by adjusting their risk perceptions, or from some other process. Having seen here that these are possibilities, we look forward to future research exploring them to characterize the basis for the relations we observed.

Communication of risk has been a difficult task for medical professionals, and our findings encourage consideration of a different approach to communicating health risks than has been typical on American cigarette packages and in other prominent health communications [58,59]. There are a large number of studies that show that the design of and warnings on cigarette packs can influence perceptions of the risks of smoking [60–68]. However, much constructive work can perhaps still be done by informing individuals about how much smoking increases their health risks. If the findings reported here are correct in suggesting that people use perceptions of relative risk when deciding whether to quit smoking, and if relative risk is indeed underestimated by most current and former smokers, corrective steps in this regard might be consequential. More specifically, if public health efforts are initiated in the future to encourage Americans to more accurately recognize the magnitudes of relative risks for various undesirable health outcomes of cigarette consumption, this may well lead to a reduction in the nation’s smoking rate and a consequent reduction in smoking-related morbidity and mortality. This may be why quantitative information about relative risk on cigarette packages in Australia (e.g., “Tobacco smoking causes more than four times the number of deaths caused by car accidents.”) appears to have been effective in encouraging smoking cessation [69].

Future research could explore these possibilities with experiments gauging the effects of different ways of describing risks on cigarette packages and other health communication mediums like television advertisements, poster campaigns, and doctor-patient communication [70]. Our findings suggest that when conducting such experiments, it may be desirable to attempt to alter people’s perceptions of relative risk in order to most directly address people’s natural approach to thinking about health risks in this arena. Perceptions of relative risk might be changed best by making such direct statements. But it may also be that such perceptions can be changed even more effectively by inducing affective reactions or in other non-quantitative ways, while simultaneously maximizing trust in the source of the information [71,72]. It is important to bear in mind that even successful efforts to change risk perceptions may not produce changes in behavior, so it will be important for future investigations to assess whether risk perception changes are translated into action [73].

In addition to their applied value, the findings reported here are interesting in basic psychological terms. By distinguishing between absolute, attributable, and relative risk, the present findings encourage future study with such measures to understand how people make many types of risky decisions and, more generally, how people trade off probabilities when making choices. And many important questions remain regarding risk perceptions involving smoking, such as how people arrive at their perceptions of relative, attributable, and absolute risk, and when and why some people use one measure rather than another to make behavioral decisions. Future studies of these sorts of issues seem merited, both in the smoking and other domains.

### Resonance with other findings

Various findings reported here resonate with findings of some past studies. For example, Viscusi [2] and Borland [69] found that people overestimated the absolute risk of smoking. Khwaja et al. [74] found that both smokers and non-smokers overestimated their risks of dying from all sorts of causes [69]. When Weinstein et al. [27] asked respondents to assess the relative risk of smoking (“Would you say the average smoker has about the same lung cancer risk as a nonsmoker, a little higher lung cancer risk than a nonsmoker, twice the nonsmoker’s risk, five times the nonsmoker’s risk, or ten times the nonsmoker’s risk?”), smokers offered underestimates.

Boney-McCoy et al. [19] found that current smokers perceived the absolute risk of smoking to be significantly lower than that perceived by former smokers. This is consistent with the evidence reported here that when considered alone, absolute risk perceptions are related to quitting in the same way. However, when controlling for relative risk, the relation of quitting to absolute risk perceptions was close to zero in the present data.

Antoñanzas et al. [75] found distributions of Spaniards’ perceptions of attributable and relative risk (regarding the impact of cigarette smoking on lung cancer and heart disease) very similar to those reported here. Viscusi et al. [76] found that each of these risk perceptions predicted Spaniards’ status as a smoker or nonsmoker when considered alone, and relative risk was a considerably stronger predictor than attributable risk, though Viscusi et al. [76] did not assess the predictive abilities of perceived attributable risk and relative risk in a single regression equation.

The present evidence that people seem to think in terms of relative risk rather than attributable or absolute risk resonates with research on effective ways to communicate risks to patients [77,78]. For example, Malenka et al. [13] asked respondents to imagine they had a disease and could choose to take one of two medications—one described in terms of its impact on relative risk (“reduces risk of dying by 80%”) and the other (statistically equivalent) described in terms of impact on attributable risk (“can prevent 8 deaths per 100 people”). Most respondents preferred the medication described in terms of relative risk, perhaps because this portrayal resonated with people’s natural way of thinking about medication benefits found that relative risk information had more impact than did attributable risk information [79–83]. These findings contrast with Saitz’s [84] and Gigerenzer et al.’s [85] speculations that people will respond as well or better to attributable risk information (presented as two absolute risks) than to relative risk information, a finding challenged by our data as well.

A preference for thinking about health risks in terms of relative risk is also apparent in news media stories. In one study, 83% of such stories reported benefits of medications in terms of relative risk only, 2% reported benefits in terms of attributable risk only, and 15% reported benefits in terms of both indicators [86]. Similarly, medical journal articles tend to focus on reports of relative risk rather than attributable risk [87].

### Other directions for further research

Future research might gain more insight into people’s natural ways of thinking about health risks by asking people to describe the health risks of smoking with whatever language they wish. With enough probing, open-ended data gathering might reveal whether people naturally use language evoking absolute risk, attributable risks, or relative risk levels, or a non-numeric representation, and such evidence is worthwhile to collect in future research [37,88]. Future work should also incorporate how much life is lost when calculating risk (see Viscusi [38] for a discussion of how this might affect an understanding of these results).

### Generalizing beyond lung cancer

The focus of the analyses reported here has been people’s perceptions of the risk of getting lung cancer due to smoking. Because lung cancer is one of the best-known health risks of smoking [11], Americans may be less likely to underestimate the relative risk of lung cancer than of other diseases that are known to be caused by smoking. If we had asked survey questions about heart disease, oral cancers, or stroke instead of lung cancer, the prevalence of underestimation of relative risk may have been even greater than was observed for lung cancer. Correcting these misunderstandings may decrease the expected smoking rate even more. Future studies can explore these possibilities.

#### Implications regarding other domains of risk perception

Differentiating perceived relative risk from perceived attributable risk may be useful in other health domains as well. For example, Meltzer and Egleston [89] reported that patients with diabetes vastly overestimated their own absolute risk of experiencing various complications. But perhaps their perceptions of relative risk are more accurate.

#### Implications for health education

Psychological research on health counseling communication has revealed errors in people’s understanding of risk information [90–92]. However, educational efforts can present risk rates in various different ways, and some presentation approaches can cause misunderstandings [93,92]. The present evidence bolsters the conclusions of some past studies suggesting that future research may be most successful when presenting relative risk information to yield better quality decisions [94–99].

## Supporting information

S1 Fig Proportions of Americans who failed to assert that smoking is dangerous to human health: Gallup Organization Surveys. (PDF)

S2 Fig Generalized Additive Models predicting the probability of being a current smoker: SRBI Survey (n = 456). (PDF)

S3 Fig Generalized Additive Models predicting the probability of being a current smoker: Harris Interactive Survey (n = 795). (PDF)

S4 Fig Generalized Additive Models predicting the probability of being a current smoker vs. former smoker: FFRISP (n = 471). (PDF)

S5 Fig Generalized Additive Models predicting the probability of being a current smoker vs. never smoker: FFRISP (n = 714). (PDF)

S1 Appendix Measuring risk. (PDF)

S2 Appendix Literature on the relation of health risk perceptions with quitting smoking. (PDF)

S3 Appendix Survey methodology. (PDF)

S4 Appendix Demographics of current and former smokers in the SRBI Survey, current and former smokers in the Harris Interactive Survey, all individuals in the FFRISP Survey, and the nation’s population. (PDF)

S5 Appendix GAMs. (PDF)

S6 Appendix Exploring responses of 500. (PDF)

S7 Appendix References for supporting information. (PDF)
