# Correction: Perceptions of health risks of cigarette smoking: A new measure reveals widespread misunderstanding

**DOI:** 10.1371/journal.pone.0212705

**Published:** 2019-02-15

**Authors:** Jon A. Krosnick, Neil Malhotra, Cecilia Hyunjung Mo, Eduardo F. Bruera, LinChiat Chang, Josh Pasek, Randall K. Thomas

The last paragraph of the “Three studies” section pertaining to ethical approval is incorrect. The correct paragraph is: The data for Study 1 were collected by the survey firm SRBI without the involvement of any academic institution, and the questionnaire was reviewed and approved by SRBI as meeting their internal ethical standards. The data for Study 2 were collected by Harris Interactive without the involvement of any academic institution, and approved by Harris Interactive as meeting their internal ethical standards. Both studies were deemed exempt, as they involved no risk to participants, and data from both studies were made available to Stanford investigators after the data collection efforts were completed. Study 3's data collection was directed by Stanford University and was approved in advance by Stanford’s IRB. All data was anonymized prior to author access, and no identifying information on the respondents was retained.
