# Supplementary material for: Perceptions of health risks of cigarette smoking: A new measure reveals widespread misunderstanding
Source: PLoS One. 2017 Aug 14;12(8):e0182063. doi: 10.1371/journal.pone.0182063 (PMC5555635; doi:10.1371/journal.pone.0182063)
Supplement: S1 Fig — (PDF) [file pone.0182063.s001.pdf]

S1 Fig. Proportions of Americans Who Failed to Assert That Smoking Is Dangerous to Human Health: Gallup Organization Surveys

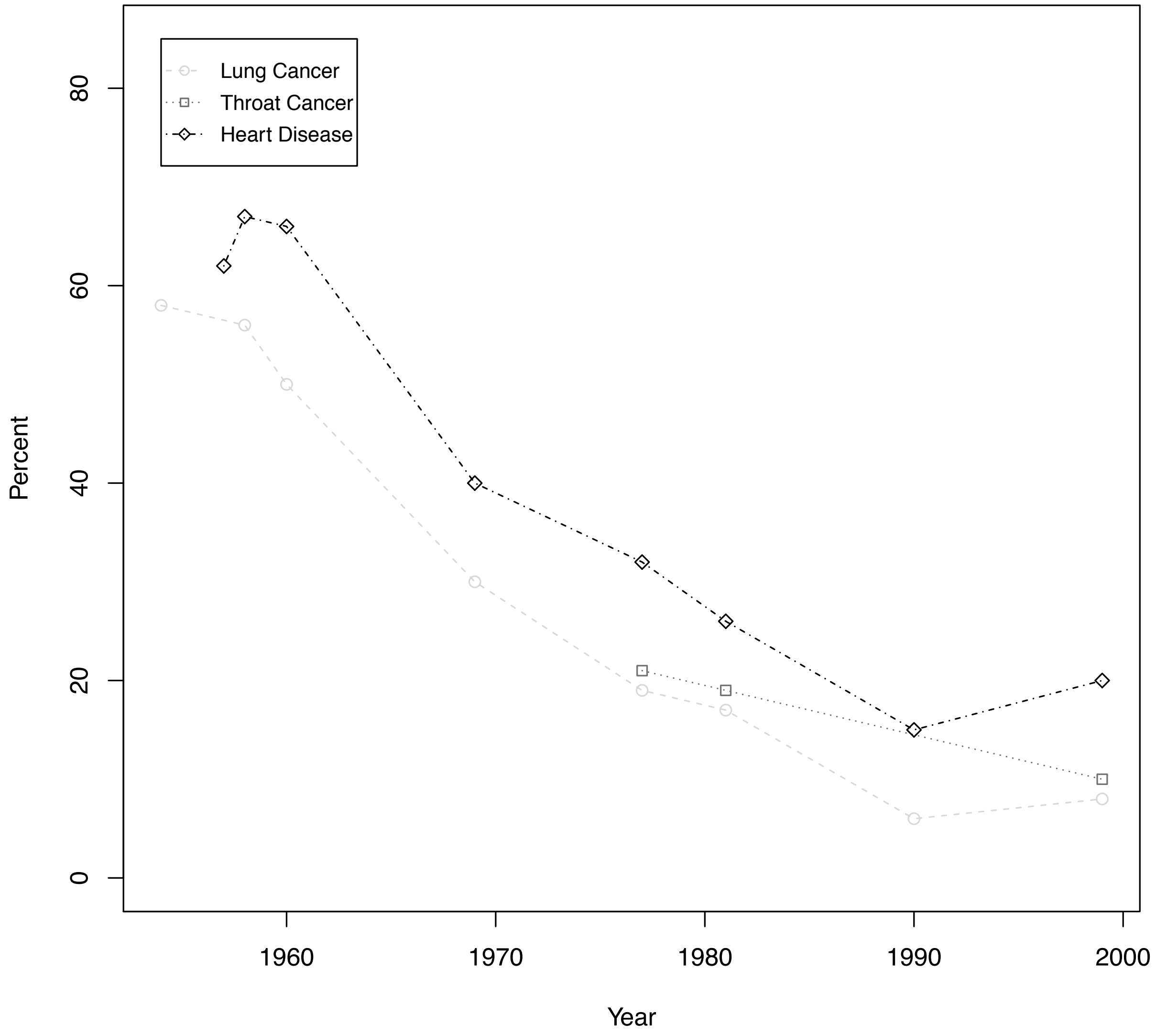

Sources: American Lung Association and Gallup Organization. (1987); Gallup Organization. (1954, 1957, 1958, 1960, 1969, 1971, 1972, 1977, 1981, 1990, 1992, 1999, 2001).
