# Supplementary material for: Perceptions of health risks of cigarette smoking: A new measure reveals widespread misunderstanding
Source: PLoS One. 2017 Aug 14;12(8):e0182063. doi: 10.1371/journal.pone.0182063 (PMC5555635; doi:10.1371/journal.pone.0182063)

S2 Fig. Generalized Additive Models Predicting the Probability of Being a Current Smoker:

SRBI Survey (n = 456)

Relative Risk vs. Attributable Risk:

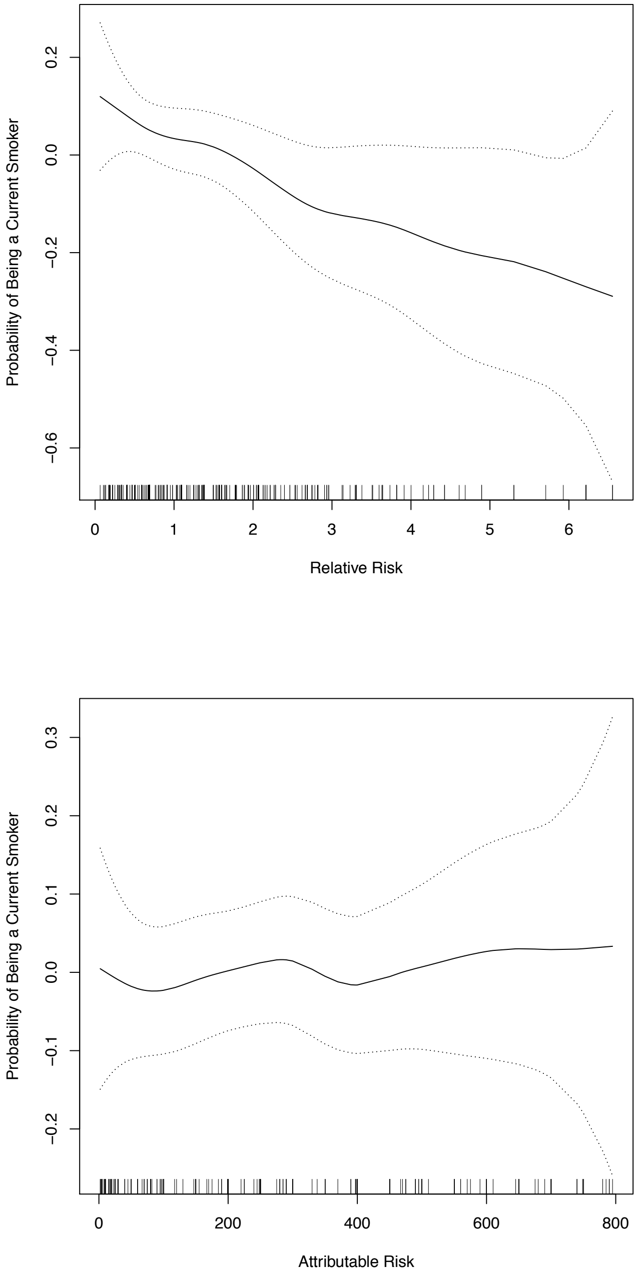

Relative Risk vs. Absolute Risk:

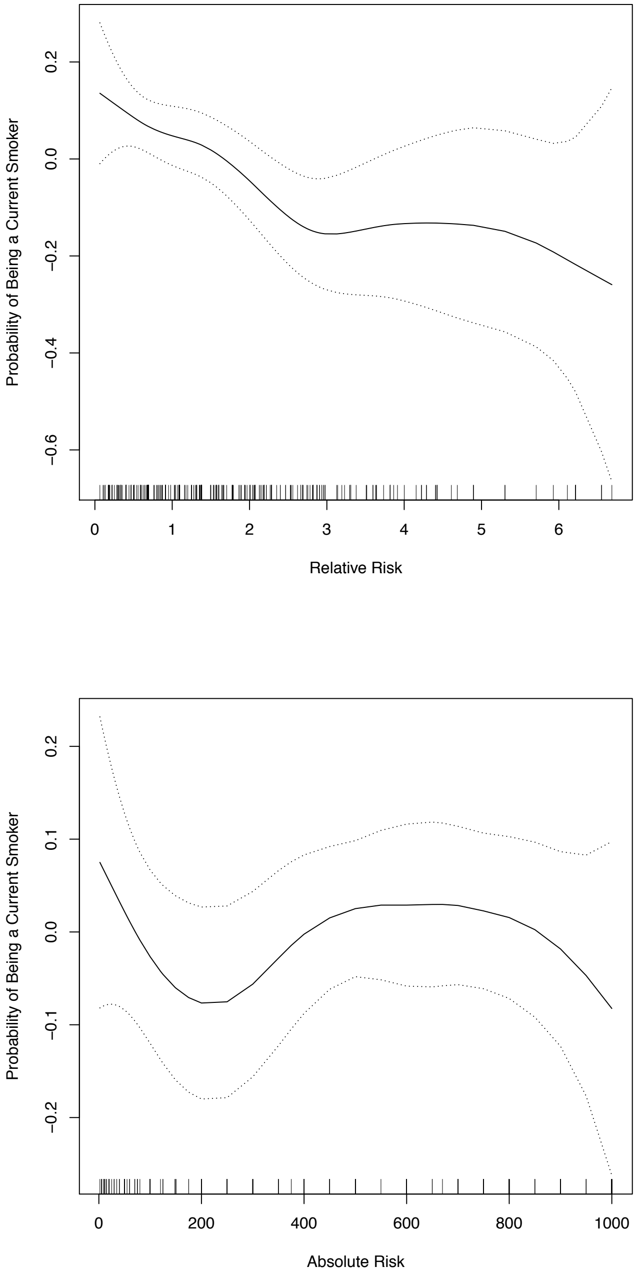

Supplement: S2 Fig — (PDF) [file pone.0182063.s002.pdf]
