# Supplementary material for: Perceptions of health risks of cigarette smoking: A new measure reveals widespread misunderstanding
Source: PLoS One. 2017 Aug 14;12(8):e0182063. doi: 10.1371/journal.pone.0182063 (PMC5555635; doi:10.1371/journal.pone.0182063)
Supplement: S1 Appendix — (PDF) [file pone.0182063.s006.pdf]

S1 Appendix: Measuring Probabilities and Numeracy

Difficulty of Mathematical Computations

The apparent use of relative risk by Americans to make decisions might seem surprising in light of past research suggesting that many people find even simple mathematical computations to be difficult to implement correctly (e.g. Batanero et al., 1994; Mokros & Russell, 1995; Reyna & Brainerd, 2007; Reyna, Nelson, Han, & Dieckmann, 2009), particularly people with less education (Galesic and Garcia-Retamero, 2010; Reyna & Brainerd, 2007) and the elderly (Galesic et al., 2009a; Reyna & Brainerd, 2007). The present findings might also seem to stand in contrast to evidence that people have more difficulty calculating ratios than they have performing addition (e.g., Anderson, 1974; Anderson & Butzin, 1974; Graesser & Anderson, 1974). Since children are typically taught addition and subtraction before multiplication and division, it might seem that subtraction (entailed in computing attributable risk) is easier than division (entailed in computing relative risk). Would not this discourage people from computing and using relative risk in their judgments and encourage them to use attributable risk instead?

In fact, once children realize that multiplication can be accomplished via repeated addition (4 x 3 = 4 + 4 + 4), they often solve multiplication problems via addition, which can entail many more computational steps but simplifies the reasoning process (see Fishbein et al., 1985, p. 3-4; see also Bell, Swan, & Taylor, 1981). Likewise, dividing 12 by 4 can be accomplished via subtraction (12 – 4 = 8, 8 – 4 = 4, 4 – 4 = 0). So in practice, multiplication and division may be no more difficult for adults than addition and subtraction. It may therefore not be so surprising that people use relative instead of attributable risk to plan their behavior, as both may be equally challenging to compute in practice.

Indeed, far simpler than computing attributable or relative risk would be to use absolute risk to make behavioral decisions. Of course, absolute risk levels alone are uninformative—without knowing the risk of lung cancer among nonsmokers, the risk among smokers does not reveal the dangers that may result from smoking. So although absolute risk is easier to conceive than are relative and attributable risk, it

should come as no surprise that people do not seem to use the former to plan their behavior.

Of course, the present investigation provides no direct evidence that people naturally compute such ratios or use them in their reasoning and behavior planning. But the present findings suggest that people behave as if they compute relative risk and use it when deciding whether to start or continue smoking. In addition to reading news about relative risk, people watch the lives of others unfold and can calculate how many smokers and nonsmokers experienced particular health problems. Some type of computation seems quite plausible. Moreover, research on numeracy has found that visual displays can be used to bridge the divide between people with high and low numeracy skills (Garcia-Retamero and Galesic 2009; Galesic et al. 2009b; Keller & Siegrist, 2009). Therefore, future research illuminating the cognitive processes people execute when generating estimates of relative risks will be useful. Such work might reveal that relative risk perceptions are indeed used more by people with better numeracy skills (see, e.g., Peters, Västfjäll, Slovic, Mertz, Mazzocco, & Dickert, 2006).

Measuring Perceived Probabilities

Since Savage (1954) asserted that perceived probabilities can only be observed indirectly (because they are revealed through the decisions people make in choice situations), many other scholars have endorsed this belief (e.g., di Finetti, 1990; Kadane & Winkler, 1988). And much literature has accumulated developing indirect methods for inferring people's perceptions of probabilities (e.g., Gilboa, 1987; Gill & Walker, 2005; Gonedes & Ijiri, 1974). But other studies have explored a variety of techniques that ask people to report perceived probabilities directly. Some have asked people to report numbers between 0% and 100% (e.g., Galanter, 1962) or to mark a graduated line from 0% to 100% (e.g., Branthwaite, 1974), or more often to use elaborate visual aids, such as bowls filled with colored balls or "probability wheels" (for reviews, see Chesley, 1975; Garthwaite, Kadane, & O'Hagan, 2005). Some studies have asked what is the "percent chance" that particular events will occur (e.g., Dominitz, 1998; Dominitz & Manski, 2004). Some observers, however, have asserted that people cannot reliably generate such reports

(see, e.g., Hogarth, 1975). The present evidence suggests that probabilities can be assessed with frequency questions, and these assessments sensibly predict actual behavior, suggesting that this assessment technique has some validity.
