# Supplementary material for: Perceptions of health risks of cigarette smoking: A new measure reveals widespread misunderstanding
Source: PLoS One. 2017 Aug 14;12(8):e0182063. doi: 10.1371/journal.pone.0182063 (PMC5555635; doi:10.1371/journal.pone.0182063)
Supplement: S2 Appendix — (PDF) [file pone.0182063.s007.pdf]

S2 Appendix: Literature on the Relation of Health Risk Perceptions with Quitting Smoking

Ex-smokers most often say that they quit because they sought improved physical health and longevity (Ahluwalia, Resnicow, & Clark, 1998); Crowe, Torabe, & Nakornkhet, 1994; Dappen, Schwartz, & O'Donnel, 1996; Duncan et al., 1992; Haaga, Gillis, & McDermut, 1993; Halpern & Warner, 1993; Lichtenstein & Cohen, 1990; Schneider, 1984; Swenson & Dalton, 1983). Also, among people who participate in a smoking cessation program, those who initially believe more strongly that smoking causes health problems are most likely to quit successfully (Kaufert et al., 1986; Tipton, 1988; c.f., Klesges et al., 1988). People who try to quit for health reasons are more likely to succeed than those who try for other reasons (Borland, 1997; Curry, Grothaus, & Wagner, 1990; Duncan et al., 1992; Halpern & Warner, 1993; c.f. Rose, Chassin, Presson, & Sherman, 1996). Schnoll et al. (2003) found that among cancer patients, smoking cessation was predicted by smoking-related risk perceptions (c.f., Clark et al., 1998; Norman, Conner & Bell, 1999). Likewise, Kreuter and Strecher (1995) found that smokers who perceived greater personal risk of stroke were more likely to quit six months later.

Other studies also suggest that beliefs about health risks are important instigators of the desire to quit. For example, Dozois, Farrow, and Miser (1995) found that 72% of a sample of current smokers attempted to quit at least once before. The reason given most often was to avoid the undesirable health consequences. Likewise, Stone and Kristeller (1992) found that smoking's deleterious health effects were the most commonly given reason by smokers for wanting to quit. Curry, Grothaus, and Wagner (1990) found that people who were motivated to quit for health reasons had a greater desire to quit than those motivated by other reasons. Several studies have found a positive correlation between personal health risk perceptions and intentions to quit (Clark et al., 1998; Norman, Conner & Bell, 1999; Weinstein, Marcus & Moser, 2005). Borland (1997) found that people who were more motivated by health concerns were more likely to intend to quit and were more likely to attempt to quit subsequently. And Rose et al. (1996) and Klesges et al. (1988) found that people who believed that smoking has deleterious health consequences

were subsequently more likely to attempt to quit (c.f., Ahluwalia, Resnicow, & Clark, 1998; Chassin, Presson, & Sherman, 1984).
