# Supplementary material for: Perceptions of health risks of cigarette smoking: A new measure reveals widespread misunderstanding
Source: PLoS One. 2017 Aug 14;12(8):e0182063. doi: 10.1371/journal.pone.0182063 (PMC5555635; doi:10.1371/journal.pone.0182063)
Supplement: S3 Appendix — (PDF) [file pone.0182063.s008.pdf]

S3 Appendix: Survey Methodology

Study One: Survey Methodology

The survey was conducted by Schulman, Ronca, and Bucuvalas, Inc. (hereafter SRBI). Random digit dialing (RDD) was conducted from a computer-assisted interviewing facility to a nationally representative sample of American adults. Interviews were conducted between August 24 and October 2, 2000. Up to 10 call-backs were made to “no answer” and “busy” telephone numbers in order to complete interviews. The adult resident of each household (age 18 or older) who either had the most recent birthday or would have a birthday next was selected to be interviewed. A total of 4,473 people were interviewed (AAPOR RR6 = 43.2%). Prior to the beginning of the interviewing, a quota of at least 2,000 interviews with current or former smokers was established.

Respondents were asked warm-up questions and then reported whether they had smoked 5 or more cigarettes during the past 7 days. Those who said “yes” were classified as current smokers. Those who said “no” were asked whether they had smoked 100 cigarettes or more during their entire lives; those who said “yes” were classified as former smokers. Forty-five percent of the 4,473 people interviewed identified themselves as current or former smokers and continued with the interview; non-smokers were asked no more questions.[1]

Some of the respondents were randomly chosen to hear and evaluate various anti-smoking messages. Here, we report results generated using only data from the 477 respondents who heard no

[1] Different researchers have defined current and former smokers in different ways (for a review, see Delnevo & Bauer, 2009; http://www.cdc.gov/NCHS/datawh/nchsdefs/cigarettesmoking.htm; accessed February 13, 2008). Since 1993, the National Health Interview Survey (NHIS) has defined former smokers as people who had smoked at least 100 cigarettes in his or her lifetimes but did not smoke now, and defined current smokers as people who smoked every day or some days at the time of an interview. Cummings et al. (2004) defined smokers as people who had smoked at least 100 cigarettes and smoked daily or on some days. Weinstein et al. (2004) defined smokers as people who had smoked at least once during the last 30 days and at least 100 cigarettes over the course of their lifetimes. Ayanian and Cleary (1999) defined a smoker as someone who smoked every day. Strecher, Kreuter, and Kobrin (1995) identified smokers as people who smoked at least one cigarette during the past 7 days. Thus, there is no single agreed-upon definition of current and former smokers. Our definition of former smokers matches that of the NHIS and those of Weinstein et al. (2004) and Cummings et al. (2004). Our definition of current smokers is narrower than those of the NHIS and Weinstein et al. (2004) but broader than those of Cummings et al. (2004), Ayanian and Cleary (1999), and Strecher, Kreuter, and Kobrin (1995).

messages. As shown in the first and third columns of Online Appendix 4, the unweighted[2] SRBI sample of 477 current and former smokers corresponds closely with the sample of current and former smokers in the 2000 National Health Interview Survey (NHIS)[3] in terms of gender, age, ethnicity, and education, though low- and high-education individuals were under-represented, and moderately-educated individuals were over-represented. Applying post-stratification weights constructed using these demographic variables made the distributions conform more closely to the NHIS (see column 4, which shows the difference between columns 2 and 3).[4]

Study Two: Survey Methodology

To assess the robustness of the SRBI survey's findings, we asked some of the same questions in a survey of a non-representative sample of current and former smokers who volunteered to complete Internet surveys in exchange for points that could be exchanged for gifts. Adult respondents were randomly selected from the Harris Interactive Internet panel (HPOL) within strata defined by sex, age, region of residence, and ethnicity. Probabilities of selection within strata were determined by probability of response (as determined by prior surveys of these groups), so the distributions of the demographics in the final sample of respondents would approximate those in the general U.S. adult population. Each panel member received an email invitation describing survey content and including a link to the survey questionnaire.

---

[2] By "unweighted," we mean that the data were weighted using the number of telephone lines that can reach the home and the number of adults living in the household to adjust for unequal probability of selection, but no post-stratification was done.

[3] The NHIS is a multi-purpose health survey conducted by the National Center for Health Statistics (NCHS), a division of the Centers for Disease Control and Prevention (CDC), and serves as the principal source of information about health in the United States (see http://www.cdc.gov/nchs/nhis.htm for more information). The U.S. Census Bureau has been the data collection agent for the NHIS, and data has been collected continuously since 1957. The NHIS is a cross-sectional household interview survey, with data collection occurring through a personal household interview by Census interviewers, and the sampling plan follows a stratified multistage probability design that permits the representative sampling of households and non-institutional group quarters (e.g., college dormitories). Both the black and Hispanic populations are oversampled. Final weights were provided to be able to adjust estimates to be nationally representative. The total household response rate of the 2000 NHIS was 88.9 percent, resulting in 32,374 adult respondents. The total household response rate of the 2006 NHIS was 87.3 percent, resulting in 24,275 adults respondents.

[4] All analyses described below weighted the data by probability of selection and implemented post-stratification. Post-stratification weights were constructed according to best practices outlined by the American National Election Studies (ANES) (DeBell & Krosnick 2009).

Respondents received a unique password and could access the survey once. Between March 16 and April 17, 2006, 16,392 participants in the HPOL database were invited to participate, and 7,847 did so (completion rate = 48%).

Respondents were asked same questions as in the SRBI survey to ascertain smoking status, and 3,967 identified themselves as current or former smokers. Some respondents were randomly chosen to read and evaluate anti-smoking messages; our focus here is on the 801 respondents who read no messages. We again constructed post-stratification weights based on sex, age, ethnicity, and education (see columns 5-8 in Online Appendix 4 for unweighted vs. weighted demographics of the survey sample and those of a representative sample of current and former smokers from the 2006 NHIS).[5]

Study Three: Survey Methodology

Our last study explored these same issues by administering the same measures to a nationally representative sample of current and former smokers and people who had never smoked via the Face-to-Face Recruited Internet Survey Platform (FFRISP). All FFRISP respondents were recruited via face-to-face area probability sampling and were given a free laptop computer (or equivalent value in cash), high-speed Internet access at home (if they did not have it already), and regular cash payments in exchange for completing monthly questionnaires for a year.

The FFRISP began with 1,000 panelists who were recruited between June and October, 2008. The smoking measures were administered during the fourth wave of data collection, initiated in January, 2009. The response rate for panel enrollment was 46% (AAPOR RR4); 973 individuals completed the fourth-wave questionnaire, yielding a 45% cumulative response rate. Weights were computed to adjust for unequal probability of selection and to post-stratify with demographics. As the last four columns in Online

[5] Even after applying capped post-stratification weights, the Harris sample of current and former smokers substantially under-represented people who had not graduated from high school. Therefore, when conducting the GAMs, we estimated a set of models controlling for education and obtained results similar to those reported in the text (see Gelman, 2007, for an argument about why this method is suitable for controlling for sample discrepancies).

Appendix 4 show, the FFRISP respondents closely resembled the American population before post-stratification and were even more similar after post-stratification.

Using the same questions as had been used in Studies 1 and 2, we found that 235 of the 973 respondents were current smokers, 222 were former smokers, and 516 had never smoked.

To test for question order effects, each respondent was randomly assigned to be asked the question about nonsmokers first or to be asked the question about smokers first. The two groups of respondents did not differ significantly from one another in terms of their answers to these questions ($p=.69$ and .62, respectively), indicating that question order had no impact on answers to these questions. Despite the lack of a significant difference, a dummy variable for question order was included in all regressions.
