# Supplementary material for: Perceptions of health risks of cigarette smoking: A new measure reveals widespread misunderstanding
Source: PLoS One. 2017 Aug 14;12(8):e0182063. doi: 10.1371/journal.pone.0182063 (PMC5555635; doi:10.1371/journal.pone.0182063)
Supplement: S4 Appendix — (PDF) [file pone.0182063.s009.pdf]

S4 Appendix: Demographics of Current and Former Smokers in the SRBI Survey, Current and Former Smokers in the Harris Interactive Survey, all individuals in the FFRISP Survey, and the Nation's Population

	SRBI (without post-stratification)	SRBI (with post-stratification)	2000 NHIS	SRBI Post-Stratified – 2000 NHIS	Interactive Survey (unweighted)	Interactive Survey (weighted)	2006 NHIS	Harris Interactive Survey	FFRISP (without post-stratification)	FFRISP (with post-stratification)	2009 CPS	FFRISP Post-Stratified – 2009 CPS
Gender												
Male	47.4%	50.0%	50.1%	-0.1%	47.1%	52.7%	52.6%	0.1%	41.9%	47.9%	48.5%	-0.6%
Female	52.6	50.0	49.9	.1	52.9	47.3	47.4	-0.1	58.1	52.1	51.5	0.6
Total %	100.0%	100.0%	100.0%		100.0%	100.0%	100.0%		100.0%	100.0%	100.0%	
Total N	477	477	54,466		801	801	10,020		968	966	207,921	
Age												
≤25	12.5%	13.3%	13.3%	0.0%	3.8%	8.3%	10.0%	-1.7%	9.7%	10.4%	12.6%	-2.2%
26-35	18.2	17.9	17.9	0.0	12.5	14.6	14.3	0.3	20.2	16.7	17.9	-1.2
36-45	19.3	22.7	22.7	0.0	15.1	16.2	17.3	-1.1	19.7	16.5	18.2	-1.7
46-55	22.2	19.2	19.2	0.0	27.8	21.9	21.3	0.5	19.2	18.9	19.5	-0.6
56-65	1.4	12.6	12.6	0.0	19.0	18.6	17.9	0.6	11.3	14.3	15.1	-0.8
66+	17.3	14.3	14.3	0.0	21.9	20.5	19.1	1.4	20.0	23.2	16.6	6.6
Total %	100.0%	100.0%	100.0%		100.0%	100.0%	100.0%		100.0%	100.0%	100.0%	
Total N	477	477	54,466		801	801	10,020		966	966	207,921	
Ethnicity [1]												
White (incl. Hispanic)	82.5%	80.2%	81.4%	-1.2%	76.9%	79.1%	86.7%	-	79.3%	80.7%	82.4%	-1.7%
(incl. Hispanic)	17.5	19.8	18.6	1.2	14.9	12.1	13.3	-	20.7	19.3	18.6	0.7
Hispanic	-	-	-		8.2	8.8	-	-	13.3	9.7	13.7	1
Total %	100.0%	100.0%	100.0%		100.0%	100.0%	100.0%		100.0%	100.0%	100.0%	
Total N	477	477	54,466		801	801	10,020		974	974	207,921	
Education												
graduate from high school	8.2%	18.2%	18.4%	-0.3%	.3%	1.3%	18.6%	-17.3%	8.3%	11.9%	14.1%	-2.2%
High school graduate	33.5	32.9	32.8	.1	22.4	40.1	32.8	7.3	24.6	31.0	30.9	0.0
college (incl. Assoc.	31.4	24.7	24.6	.1	45.9	32.3	26.7	5.6	38.3	31.0	28.0	2.9
Bachelor's degree	16.3	17.1	17.1	.1	19.3	18.7	15.5	3.1	17.2	15.4	17.7	-2.4
School	1.6	7.1	7.1	.0	12.1	7.7	6.4	1.3	11.5	11.1	9.3	2.7
Total %	100.0%	100.0%	100.0%		100.0%	100.0%	100.0%		100.0%	100.0%	100.0%	
Total N	477	477	52,005		801	801	9,891		971	970	207,921	

1. Ethnicity was measured differently in the Harris and NHIS studies, precluding direct comparability. Nevertheless, even the unweighted percentages of non-whites closely approximated the NHIS sample.
