# Supplementary material for: Perceptions of health risks of cigarette smoking: A new measure reveals widespread misunderstanding
Source: PLoS One. 2017 Aug 14;12(8):e0182063. doi: 10.1371/journal.pone.0182063 (PMC5555635; doi:10.1371/journal.pone.0182063)
Supplement: S5 Appendix — (PDF) [file pone.0182063.s010.pdf]

S5 Appendix: GAMs

A GAM is similar to a logistic regression, but the former does not assume a linear relation between risk perceptions and quitting. Instead, quitting is predicted by an additive model including several non-parametric functions (for technical details, see Hastie & Tibshirani, 1990). Estimating a logistic GAM is preferred to standard parametric regression techniques because relaxing the assumption of linearity prevents model misspecification. Inspection of the raw data revealed nonlinear relations of relative, attributable, and absolute risks with quitting. Because mis-specifying a model by imposing linearity can substantially distort inferences, especially when two variables are highly correlated, the flexibility and weak assumptions of the GAM are preferable. In the SRBI data, the distribution of relative risk was substantially skewed (skewness=6.40), whereas the distributions of attributable risk (skewness = .59) and absolute risk (skewness = .06) were not, so relative risk was logged for the analyses. The GAM results reported here were generated without including any demographic controls. When we re-estimated the GAM parameters controlling for demographics, the results obtained were comparable to those reported in the text.
