# Supplementary material for: Perceptions of health risks of cigarette smoking: A new measure reveals widespread misunderstanding
Source: PLoS One. 2017 Aug 14;12(8):e0182063. doi: 10.1371/journal.pone.0182063 (PMC5555635; doi:10.1371/journal.pone.0182063)
Supplement: S6 Appendix — (PDF) [file pone.0182063.s011.pdf]

S6 Appendix: Exploring Responses of 500

There may be an illusion hidden in these results, as suggested by the work of Fischhoff and his colleagues (Bruine de Bruin, Fischhoff, Millstein, & Halpern-Felsher, 2000; Fischhoff & Bruine de Bruin, 1999), who argued that when people are asked to report a probability but do not know the answer, they sometimes answer “50,” meaning “fifty-fifty” or “unknown,” rather than meaning a 50% chance. To explore the impact of this potential source of measurement error on our conclusions, we re-estimated the logistic GAM after dropping the respondents who answered “500” to the question about nonsmokers or to the question about smokers. After sub-setting the data in this manner, the resulting parameter estimates supported the same conclusions even more strongly.

When comparing relative risk to attributable risk, the increase in the probability of quitting from moving across the interquartile range of relative risk was 16.2%. In contrast, moving across the interquartile range of attributable risk increased the probability of quitting by only 2.4%. Adding relative risk to a model predicting quitting with attributable risk significantly improved fit (p=.007). But adding attributable risk to a model predicting quitting with relative risk again failed to significantly improve fit (p=.45). Adding relative risk to a model with only absolute risk improved fit significantly (p=.001), whereas adding absolute risk to a model with only relative risk had no significant impact on fit (p=.17). Movement across the interquartile range of relative risk in this analysis produced an increase in the probability of quitting of 18.9%; movement across the same range for absolute risk decreased the probability of quitting by 8.8%.

Another way to handle the respondents who answered “500” to questions about nonsmokers or smokers would be to impute values for these respondents via multiple imputation generated using iterative predictive models (Rubin, 1987; Schafer, 1997; Schafer & Olsen, 1998). We did so using all available survey variables as predictors, including general perceptions of the quality of health care that most Americans received, how good a job President Clinton was doing to handle health care in the country, current smoking status, other measures of beliefs about smoking and lung cancer, the extent of regret and

negative feelings due to smoking, race, age, and education. Data augmentation was conducted with 50 iterations (k=50) to generate 5 imputations (m=5). Because the highest proportion of missing values was 23%, efficiency of estimates based on 5 imputations was 95.6% (Rubin, 1987). We averaged the values obtained by the imputations to yield a set of replacements for the 500s that respondents reported.

When we re-estimated the logistic GAMs with the imputed data, the results were similar to those reported above. Adding relative risk as a predictor to a model predicting quitting with attributable risk significantly improved fit (p=.008). Adding attributable risk as a predictor to a model predicting quitting with relative risk again did not improve fit significantly (p=.55). In a model predicting quitting with relative and attributable risk, movement across the interquartile range of relative risk was associated with a 12.7% increase in the probability of quitting. Movement across the interquartile range of attributable risk produced a 1.3% decrease in likelihood of quitting.

Adding relative risk to a model with only absolute risk again improved model fit significantly (p=.001). The addition of absolute risk to a model with relative risk did not significantly improve the fit (p=.32). As expected, movement across the interquartile range of relative risk was associated with a 14.5% increase in the probability of quitting, whereas movement across the interquartile range of absolute risk produced a 3.0% decrease in the likelihood of quitting.

Last, we dropped the respondents who gave a rating of 500, and used these respondents' imputed values instead of their 500s (see columns 3 and 4 of each table). Dropping the 500s or imputing the values did not change the summary statistics substantially and did not support different conclusions about the prevalence of overestimation and underestimation of risk. Likewise, in Study Two, dropping respondents who answered "500" to either survey question produced comparable results.

In Study Three, the respondents who said that 500 of the 1000 smokers would get lung cancer (14.3%) or that 500 of the 1000 nonsmokers would get lung cancer (3.9%) were asked: "Did you type 500 because you don't know, or because you think about half of the 1,000 people would get lung cancer?"

People who said “don’t know” (5.4% for smokers and 1.9% for nonsmokers) were asked, “If you had to guess, about how many of those 1,000 people do you think would get lung cancer sometime during their lives?” When a person’s response to this follow-up differed from his or her original answer, the second response replaced the initial answer for our analyses. Because we asked these follow-up questions, there was no need to drop or impute people who selected “500” for Study 3.
