# Supplementary material for: Perceptions of health risks of cigarette smoking: A new measure reveals widespread misunderstanding
Source: PLoS One. 2017 Aug 14;12(8):e0182063. doi: 10.1371/journal.pone.0182063 (PMC5555635; doi:10.1371/journal.pone.0182063)
Supplement: S7 Appendix — (PDF) [file pone.0182063.s012.pdf]

S7 Appendix: References for Supporting Information

Ahluwalia, J. S., Resnicow, K., & Clark, W. S. (1998). Knowledge about smoking, reasons for smoking, and reasons for wishing to quit in inner-city African Americans. *Ethnicity Dis*., *8*, 385-393.

American Lung Association and Gallup Organization. (1987). National survey conducted June, 1987. *iPOLL Databank*, *The Roper Center for Public Opinion Research, University of Connecticut*. Retrieved February 27, 2008 from http://www.ropercenter.uconn.edu/ ipoll.html

Anderson, N. H. (1974). Cognitive algebra: Integration theory applied to social attribution. *Advances in Experimental Social Psychology*, 7, 1-101.

Anderson, N. H., & Butzin, C.A. (1974). Performance = motivation x ability: An integration-theoretical analysis. *Journal of Personality and Social Psychology*, *30*, 598-604.

Ayanian, J. Z., & Cleary, P. D. (1999). Perceived risks of heart disease and cancer among cigarette smokers. *Journal of the American Medical Association*, *281*, 1019-1021.

Batanero, C., Godino, J. D., Vallecillos, A., Green, D. R., & Holmes, P. (1994). Errors and difficulties in understanding elementary statistical concepts. *International Journal of Mathematics Education in Science and Technology*, *25*, 527-547.

Bell, A., Swan, M., & Taylor, G. (1981). Choice of operations in verbal problems with decimal numbers. *Educational Studies in Mathematics*, *12*, 399-420.

Borland, R. (1997). What do people's estimates of smoking related risk mean. *Psychological Health*, *12*, 513-521.

Branthwaite, A. (1974). A note comparing three measures of subjective probability, their validity and reliability. *Acta Psychologica*, *38*, 337-342.

Bruine de Bruin, W., Fischhoff, B., Millstein, S. G., & Halpern-Felsher, B. L. (2000). Verbal and numerical expressions of probability: "It's a fifty-fifty chance." *Organizational Behavior and Human Decision*

Processes, *81*, 115-131.

Chassin, L., Presson, C. C., & Sherman, S. J. (1984). Cognitive and social influence factors in adolescent smoking cessation. *Addictive Behaviors*, *9*, 383-390.

Chesley, G. R. (1975). Elicitation of subjective probabilities: A review*. The Accounting Review*, *50*, 325-337.

Clark, M. A., Kviz, F. J., Crittenden, K. S., & Warnecke, R. B. (1998). Psychosocial factors and smoking cessation behaviors among smokers who have and have not ever tried to quit. *Health Education Research*, *13*, 145-153.

Crowe, J. W., Torabi, M. R., & Nakornkhet, N. (1994). Cross-cultural study of samples of adolescents' attitudes, knowledge, and behaviors related to smoking. *Psychological Reports*, *75*, 1155-1161.

Cummings K. M., Hyland, A, Giovino, G. A., Hastrup, J., Bauer, J., & Bansal, M. A. (2004). Are smokers adequately informed about the health risks of smoking and medicinal nicotine? *Nicotine & Tobacco Research*, *6(Supplement 3)*, S333-S340.

Curry, S., Grothaus, L. C., & Wagner, E. H. (1990). Intrinsic and extrinsic motivation for smoking cessation. *Journal of Consulting and Clinical Psychology, 58,* 310-316.

Dappen, A., Schwartz, R. H., & O'Donnel, R. (1996). A survey of adolescent smoking patterns. *Journal of the American Board of Family Practice*, *9*, 7-13.

DeBell, M., & J.A. Krosnick. (2009). Computing weights for American National Election Study survey data. *Ann Arbor, MI, and Palo Alto, CA: American National Election Studies.*

Di Finetti, B. (1990). *Theory of probability*. New York: Wiley.

Delnevo, C. D., & Bauer, U. E. (2009). Monitoring the tobacco use epidemic III: The host: Data sources and methodological challenges. *Preventive Medicine*, *48*, S16-S23.

Dominitz, J. (1998). Earnings expectations, revisions and realizations. *Review of Economics and Statistics*, *80*, 374-388.

Dominitz, J., & Manski, C. F. (2004). How should we measure consumer confidence? *The Journal of Economic Perspectives*, *18*, 51-66.

Dozois, D. N., Farrow, J. A., & Miser, A. (1995). Smoking patterns and cessation motivations during adolescence. *International Journal of the Addictions*, *30*, 1485-1498.

Duncan, C. L., Cummings, S. R., Hudes, E. S., Zahnd, E., & Coates, T. J. (1992). Quitting smoking: Reasons for quitting and predictors of cessation among medical patients. *Journal of General Internal Medicine*, *7*, 398-404.

Fischhoff, B., & Bruine de Bruin, W. (1999). Fifty-fifty = 50%? *Journal of Behavioral Decision Making*, *12*, 149-163.

Fishbein, E., Deri, M., Nello, M. S., & Marino, M. S. (1985). The role implicit models in solving verbal problems in multiplication and division. *Journal for Research in Mathematics Education*, *16*, 3-17.

Garcia-Retamero, R, & Galesic M. (2009). Communicating risk reduction to people with low numeracy skills: a cross-cultural comparison. *American Journal of Public Health*, *99*, 2196-2202.

Galanter, E. (1962). The direct measurement of utility and subjective probability. *The American Journal of Psychology*, *75*, 208-220.

Galesic, M., & Garcia-Retamero, R. (2010). Statistical numeracy for health: a cross-cultural comparison with probabilistic national samples. *Archives of Internal Medicine*, *170*, 462-68.

Galesic, M., Gigerenzer, G., & Straubinger, N. (2009a) Natural frequencies help older adults and people with low numeracy to evaluate medical screen tests. *Medical Decision Making*, *29*, 368-371.

Galesic, M., Garcia-Retamero, R., & Gigerenzer, G. (2009b). Using icon arrays to communicate medical risks to low-numeracy people. *Health Psychology, 28,* 210-216.

Gallup Organization. (1954, 1957, 1958, 1960, 1969, 1971, 1972, 1977, 1981, 1990, 1992, 1999, 2001). National survey. *iPOLL Databank, The Roper Center for Public Opinion Research, University of Connecticut.* Retrieved August 6, 2010 and February 27, 2008 from

http://www.ropercenter.uconn.edu/ipoll.html

Garthwaite, P. H., Kadane, J. B., & O'Hagan, A. (2005). Statistical methods for eliciting probability distributions. *Journal of the American Statistical Association*, *100*, 680-700.

Gelman, A. (2007). Struggles with survey weighting and regression modeling. *Statistical Science*, *2*, 153-164.

Gill, J., & Walker, L. D. (2005). Elicited priors for Bayesian model specifications in political science research. *Journal of Politics*, *67*, 841-872.

Gilboa, I. (1987). Expected utility with purely subjective non-additive probabilities. *Journal of Mathematical Economics*, *16*, 65-88.

Gonedes, N. J., & Ijiri, Y. (1974). Improving subjective probability assessment for planning and control in team-like organizations. *Journal of Accounting Research*, *12*, 251-269.

Graesser, C. C., and Anderson, N. H. (1974). Cognitive algebra of the equation: Gift size = generosity x income. *Journal of Experimental Psychology*, 103, 692-699.

Haaga, D., Gillis, M. M., & McDermut, W. (1993). Lay beliefs about the causes and consequences of smoking cessation maintenance. *International Journal of the Addictions*, *28*, 369-375.

Halpern, M. T., & Warner, K. E. (1993). Motivations for smoking cessation: A comparison of successful quitters and failures. *Journal of Substance Abuse, 5*, 247-256.

Hastie, T., & Tibishirani, R. (1990). *Generalized Additive Models*. London: Chapman and Hall.

Hogarth, R. M. (1975). Cognitive processes and the assessment of subjective probability Distributions. *Journal of the American Statistical Association*, *70*, 271-289.

Kadane, J. B., & Winkler, R. L. (1988). Separating probability elicitation from utilities. *Journal of the American Statistical Association, 83*, 357-363.

Kaufert, J. M., Rabkin, S. W., Syrotuik, J., Boyko, E., & Shane, F. (1986). Health beliefs as predictors of

success of alternate modalities of smoking cessation: Results of a controlled trial. *Journal of Behavioral Medicine*, *9*, 475-489.

Keller, C., & Siegrist, M. (2009). Effect of risk communication formats on risk perception depending on numeracy. *Medical Decision Making*, *29*, 483-490.

Klesges, R. C., Somes, G., Pascale, R. W., Klesges, L. M., Murphy, M., Brown, K., & Williams, E. (1988). Knowledge and beliefs regarding the consequences of cigarette smoking and their relationships to smoking status in a biracial sample. *Health Psychology*, *7*, 387-401.

Kreuter, M. W., & Strecher, V. J. (1995). Changing inaccurate perceptions of health risks: Results from a randomized trial. *Health Psychology*, *14*, 56-63.

Lichtenstein, E., & Cohen, S. (1990). Prospective analysis of two modes of unaided smoking cessation. *Health Education Research*, *5*, 63-72.

Mokros, L., & Russell, S. J. (1995). Children's concepts of average and representativeness. *Journal for Research in Mathematics Education*, *26*, 20-39.

Norman, P., Conner, M., & Bell. R. (1999). The theory of planned behavior and smoking cessation. *Health Psychology*, *18*, 89-94.

Peters, E., Västfjäll, D., Slovic, P., Mertz, C.K., Mazzocco, K., & Dickert, S. (2006). Numeracy and decision making. *Psychological Science*, *17*, 407-413.

Reyna, V. F., & Brainerd, C. J. (2007). The importance of mathematics in health and human judgment: Numeracy, risk communication, and medical decision making. *Learning and Individual Differences*, *17*, 147-159.

Reyna, V. F., Nelson, W. L., Han, P. K., & Dieckman, N. F. (2009). How numeracy influences risk comprehension and medical decision making. *Psychological Bulletin*, *135*, 943-973.

Rose, J. S., Chassin, L., Presson, C. C., & Sherman, S. J. (1996). Prospective predictors of quit attempts and smoking cessation in young adults. *Health Psychology*, *15*, 261-268.

Rubin, D. B. (1987). *Multiple imputation for nonresponse in surveys*. New York: Wiley.

Savage, L. J. (1954). Elicitation of personal probabilities and expectations. *Journal of the American Statistical Association*, *66*, 783-801.

Schafer, J. L. (1997). *Analysis of incomplete multivariate data*. London: Chapman & Hall.

Schafer, J. L., & Olsen, M. K. (1998). Multiple imputation for multivariate missing-data problems: A data analyst's perspective. Unpublished manuscript.

Schneider, S. J. (1984). Who quits smoking in a behavioral treatment program? *Addictive Behaviors*, *9*, 373-381.

Schnoll, R. A., James, C., Malstrom, M.., Rothman, R. L., Wang, H., Babb, J., et al. (2003). Longitudinal predictors of continued tobacco use among patients diagnosed with cancer. *Annals of Behavioral Medicine*, *25*, 214-221.

Strecher, V. J., Kreuter, M. W., & Kobrin, S. C. (1995). Do cigarette smokers have unrealistic perceptions of their heart attack, cancer, and stroke risks? *Journal of Behavioral Medicine*, *18*, 45-54.

Stone, S. L., & Kristeller, J. L. (1992). Attitudes of adolescents towards smoking cessation. *American Journal of Preventive Medicine*, *8*, 221-225.

Swenson, I., & Dalton, J. A. (1983). Reasons for smoking cessation among a random sample of North Carolina nurses. *Women and Health*, *8*, 33-41.

Tipton, R.M. (1988). The effects of beliefs about smoking and health on smoking cessation. *Journal of Psychology*, *122*, 313-21.

Weinstein, N. D., Slovic, P., Waters, E., & Gibson, G. (2004). Accuracy and optimism in smokers' beliefs about quitting. *Nicotine and Tobacco Research*, *6*, 349-355

Weinstein, N. D., Marcus, S. E., & Moser, R. P. (2005). Smokers' unrealistic optimism about their risk. *Tobacco Control*, *14*, 55-59.
